# B-STARS2: Early contralesional continuous theta burst stimulation (cTBS) to promote upper limb recovery after stroke – Rationale and design of a phase-3 multicentre, randomised, sham-controlled, clinical trial

**DOI:** 10.1177/23969873251355937

**Published:** 2025-07-08

**Authors:** Jord JT Vink, Tessa A Verhoeff, Willem M Otte, Miriam P van der Meulen, H Bart van der Worp, Johanna MA Visser-Meily, Rick M Dijkhuizen

**Affiliations:** 1 Translational Neuroimaging Group, Center for Image Sciences, University Medical Center Utrecht and Utrecht University, Utrecht, The Netherlands; 2 Center of Excellence in Rehabilitation Medicine, University Medical Center Utrecht Brain Center, University Medical Center Utrecht, Utrecht University and De Hoogstraat Rehabilitation, Utrecht, The Netherlands; 3 Department of Pediatric Neurology, UMC Utrecht Brain Center, University Medical Center Utrecht and Utrecht University, Utrecht, The Netherlands; 4 Julius Center for Health Sciences and Primary Care, Utrecht University, Utrecht, The Netherlands; 5 Department of Neurology and Neurosurgery, UMC Utrecht Brain Center, University Medical Center Utrecht and Utrecht University, Utrecht, The Netherlands; 6 Department of Rehabilitation, Physical Therapy Science and Sports, Brain Center, University Medical Center Utrecht and Utrecht University, Utrecht, The Netherlands

**Keywords:** Stroke, upper limb recovery, continuous theta burst stimulation, rTMS, cTBS, brain stimulation, repetitive transcranial magnetic stimulation, randomised controlled trial, rehabilitation

## Abstract

**Rationale:**

Many stroke survivors have persisting upper limb impairment. In a phase-2 trial, early contralesional continuous theta burst stimulation (cTBS) improved upper limb recovery and functional outcome after stroke, but confirmation of this benefit in a larger, phase-3 trial is required before this can be recommended as standard of care.

**Aim:**

We aim to assess whether 10 sessions of cTBS of the contralesional primary motor cortex, combined with regular care upper limb training, started within 3 weeks after stroke onset and continued for 2 weeks, reduces upper limb impairment at 90 days after stroke as compared to sham stimulation.

**Methods and design:**

We will perform a multicentre, double-blind, randomised, sham-controlled, clinical trial. Patients with ischaemic stroke or intracerebral haemorrhage and unilateral upper limb paresis will be assigned to receive 10 daily sessions of active or sham cTBS, delivered over the contralesional primary motor cortex, combined with regular care upper limb therapy and started within 3 weeks after stroke onset.

**Outcomes:**

The primary outcome is the score of the Fugl-Meyer Upper Extremity (FM-UE) assessment at 90 days after stroke. Secondary outcomes are the FM-UE score at 12 months after stroke and scores on the Action Research Arm Test, Nine Hole Peg Test, modified Rankin Scale, Barthel Index, hand, participation and overall recovery sections of the Stroke Impact Scale and the EuroQol-5D-5L at 90 days and 12 months post-stroke. Additionally, cost-effectiveness, length of stay at the rehabilitation centre, and ipsilesional and contralesional excitability are assessed.

**Sample size:**

We will randomise 454 participants 1:1 to active or sham cTBS. The sample size is based on a minimal detectable effect of 6.6 points on the FM-UE scale.

**Discussion:**

If cTBS treatment leads to a cost-effective and clinically meaningful additional recovery of at least 6.6 points on the FM-UE scale at 90 days after stroke, then cTBS treatment can be recommended as standard of care.

## Graphical abstract

**Graphical abstract:**
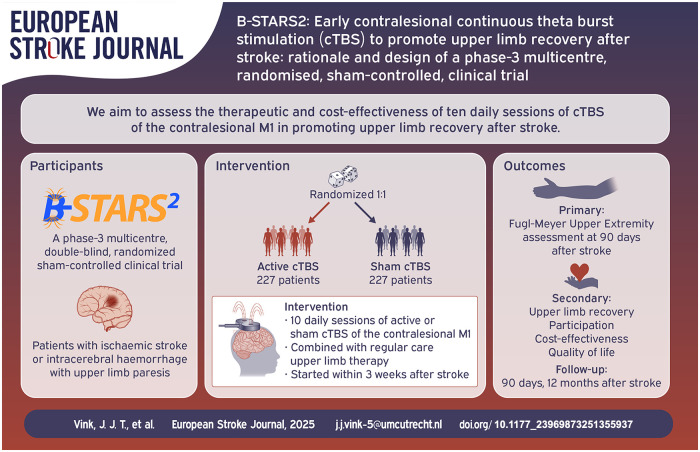

## Introduction and rationale

Despite advances in acute stroke treatment, many stroke survivors are left with disabling upper limb impairment.^1^ The current standard of care for post-stroke upper limb impairment is intensive rehabilitation, which consists primarily of training-based therapies, such as physical and occupational therapy. These therapies often fail to achieve a full recovery, hampering activities of daily living, societal participation and quality of life.^2,3^

Patients with post-stroke motor deficits have been shown to have an increased inhibitory drive from the contralesional to the ipsilesional primary motor cortex (M1) as compared to the other way round.^4,5^ It has been postulated that this interhemispheric imbalance may be counterbalanced by inhibition of the contralesional M1, resulting in a brain state that is more prone to spontaneous and therapy-induced recovery.^6^

In recent years, non-invasive brain stimulation techniques, such as repetitive transcranial magnetic stimulation (rTMS), have shown promise as a therapeutic means to modulate brain activity and promote functional recovery after stroke.^7^ This applies in particular to inhibitory forms of rTMS, such as low-frequency and continuous theta burst stimulation (cTBS), when applied to the contralesional M1 during the first month after stroke.^8,9^ In a recent single-centre phase-2 randomised trial in 60 patients with ischaemic stroke or intracerebral haemorrhage, 10 sessions of an inhibitory form of repetitive transcranial magnetic stimulation (rTMS), that is, continuous theta burst stimulation (cTBS), applied to the contralesional primary motor cortex (M1), started within 3 weeks after stroke, led to an additional upper limb recovery of over 9 points on the Fugl-Meyer Upper Extremity (FM-UE) score, better functional recovery and a reduction in the time spent in a rehabilitation centre of 18 days.^10^ These promising findings need to be replicated in a larger phase-3 trial before guidelines can recommend rTMS treatment for the promotion of upper limb recovery as standard of care.

We therefore aim to assess the therapeutic effectiveness and cost-effectiveness of 10 daily sessions of cTBS of the contralesional M1, combined with regular care upper limb training and started within 3 weeks after stroke onset, in promoting upper limb recovery after stroke.

## Methods

### Study design

**Figure 1.**
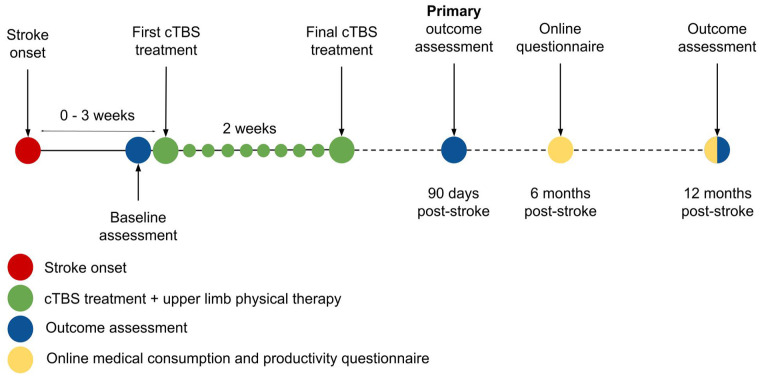
Study timeline.

We will perform a phase-3 multicentre, double-blind, randomised (1:1), sham-controlled clinical trial, named B-STARS2. Stroke patients will be recruited from 16 rehabilitation centres in the Netherlands and will be assigned randomly to receive 10 daily sessions of active or sham cTBS, delivered over the contralesional M1 and combined with regular care upper limb therapy. The active comparison is active versus sham cTBS. The duration of patient inclusion is 4 years and has started in December 2024. The total study duration is 5 years (Figure 1).

### Participant population

Patients aged 18 years or older with a first-ever ischaemic stroke or intracerebral haemorrhage within a cerebral hemisphere or the brainstem and a unilateral upper limb paresis with a motricity index between 9 and 99, who can start treatment within 21 days after stroke onset and can provide written informed consent are eligible to participate. Exclusion criteria are upper limb paresis prior to stroke onset, an absolute contra-indication to TMS, incapacity or severe impairments that can impede study participation as determined by the treating rehabilitation physician (e.g. extreme fatigue or severe communication deficits) and a life expectancy shorter than 1 year. Absolute contra-indications to TMS are magnetically sensitive objects implanted in or guided through the head or neck area (e.g. cochlear implants, implanted neurostimulator, pacemaker or defibrillator leads, metal fragments, or metal clips) with the exception of dental work (e.g. fillings, braces, or implants), a history of epilepsy, pregnancy or other contra-indications that may potentially be harmful as determined by the treating rehabilitation physician.

### Randomisation and blinding

Participants are randomly assigned in a 1:1 ratio to one of the two treatment groups (group A or B) using a variable block randomisation model that is implemented in a web-based data management service (CASTOR). Treatment allocation is stratified by rehabilitation centre (stratification factor 1) and the ability to extend one or more fingers of the affected hand (stratification factor 2).

Sham cTBS will be performed using a sham TMS coil, which looks, sounds and operates identically to an active TMS coil but has magnetic shielding or altered coil geometry, preventing effective stimulation. The sham and active TMS coils will be labelled with a code (A or B) by a coordinating investigator who is not involved in the TMS treatments, concealing true treatment allocation from therapists who perform randomisation and TMS treatment, ensuring a double-blind study design. For resting motor threshold (RMT) determination, a separate TMS coil (labelled RMT coil) will be used, to conceal the nature of the active and sham coil during cTBS treatment preparation. After the final cTBS session, participants are asked whether they think they received active or sham cTBS to assess blinding integrity.

### Treatment

The intervention consists of 10 daily sessions of active or sham cTBS, delivered by trained therapists over the contralesional M1 in a period of 10 consecutive working days within a maximum period of 17 days. The first cTBS session takes place within 21 days after stroke onset. cTBS treatment will be delivered prior to at least 40 min of regular care upper limb therapy, with a maximum time interval between cTBS and upper limb therapy of 15 min. Regular care upper limb therapy will be performed according to local protocols, which are based on national guidelines from the royal Dutch Society for Physical Therapy and will be adjusted based on the patient’s level of upper limb impairment. TMS procedures will be performed in accordance with recent safety guidelines for TMS.^11^

The cTBS target location is defined as the position on the scalp at which motor evoked potentials (MEPs) with the largest peak-to-peak amplitude could be evoked in the first dorsal interosseous (FDI) muscle of the unaffected arm. The target location is identified by delivering TMS pulses with the RMT coil while monitoring the electromyogram (EMG) and its coordinates are stored in a neuronavigation system. Stimulation intensity is set at 70% of the RMT, which is defined as the minimum machine output at which stimulation evokes at least 5 of 10 MEPs with a peak-to-peak amplitude of over 50 μV with the TMS coil oriented 45° to the midline and an interstimulus interval of 7 s.

cTBS consists of continuous delivery of bursts of three biphasic stimuli at 50 Hz repeated at five bursts per second for a duration of 40 s resulting in a total of 600 stimuli. During treatment, the TMS coil is placed on the scalp over the target location and oriented at 45° to the midline. cTBS treatment will be performed according to international TMS guidelines.^11^

### Primary outcome

The primary outcome is the FM-UE score at 90 days post-stroke. The FM-UE is a reliable and valid motor performance test consisting of 33 tasks that assesses upper limb motor impairment on a scale from 0 to 66 points, with a higher score indicating less impairment.^12^ The FM-UE will be assessed by certified assessors, who have received on-site training by the team of coordinating investigators and have successfully completed an FM-UE exam.

### Secondary outcomes

Secondary outcomes are the score on the FM-UE at 12 months post-stroke and the scores on the Action Research Arm Test (ARAT), Nine Hole Peg Test (NHPT), modified Rankin Scale (mRS), Barthel Index (BI), hand, participation and overall recovery sections of the Stroke Impact Scale (SIS-Hand, SIS-Participation and SIS-Recovery) and the EuroQol-5D-5L at 90 days and 12 months post-stroke (Table 1). Other secondary outcomes are the EuroQol-5D-5L at 6 months post-stroke, length of stay at the rehabilitation centre, change in ipsilesional and contralesional excitability and cost effectiveness.

**Table 1.** Outcome measurements.

Assessment	Timepoint	Description
Fugl-Meyer Assessment – Upper Extremity	Baseline, 90 days, 12 months	Upper extremity impairment test
Action Research Arm Test	Baseline, 90 days, 12 months	Upper extremity activity test
Nine Hole Peg Test	Baseline, 90 days, 12 months	Manual dexterity test
Modified Rankin Scale	Baseline, 90 days, 12 months	Disability and dependence questionnaire
Barthel Index	Baseline, 90 days, 12 months	Activities of daily living questionnaire
Stroke Impact Scale – Hand	Baseline, 90 days, 12 months	Hand function questionnaire
Stroke Impact Scale - Participation	Baseline, 90 days, 12 months	Participation questionnaire
Stroke Impact Scale – Overall recovery	Baseline, 90 days, 12 months	Self-reported recovery on a scale from 0 to 100
EuroQol–5D-5L	Baseline, 90 days, 6 and 12 months	Quality of life questionnaire
Length of stay		Length of stay at the rehabilitation centre
Ipsilesional excitability	Before the 1st and after the 10th cTBS session	Resting motor threshold of the ipsilesional primary motor cortex
Contralesional excitability	Before the 1st, 6th and 10th cTBS session	Resting motor threshold of the contralesional primary motor cortex
Contralesional inhibition	Before and after the 1st, 6th and 10th cTBS session	Change in the average motor-evoked potential amplitude before and after cTBS.

cTBS: continuous theta burst stimulation.

#### Action research arm test (ARAT)

The ARAT is an upper limb performance test which assesses the ability to perform gross movements and the ability to grasp, move and release objects differing in size, weight and shape.^13^ The test consists of 19 tasks on a scale from 0 to 57 points, with a higher score indicating better performance.

#### Nine hole peg test (NHPT)

The NHPT is a reliable and valid performance test that examines fine motor skills and manual dexterity.^14^ Nine pegs have to be placed into slots and individually placed back into a bin with the affected hand. The main outcome is duration, with a maximum duration of 50 s. A secondary outcome is the number of pegs that have been transferred successfully (within the 50 s time window), with a maximum of 18 points (1 point per peg movement).

#### Modified Rankin Scale (mRS)

The mRS is a measure of the degree of disability and dependence on an ordinal scale from 0 to 6, with a lower score indicating less disability and dependence. The mRS will be assessed through a structured interview, increasing its reliability.^15^

#### Barthel index (BI)

The BI is a measure of independence assessing performance of 10 activities of daily living on an ordinal scale from 0 to 100 points (5-point increments), with a higher score indicating better performance.^16^

#### Stroke impact scale (SIS)

The Stroke Impact Scale (SIS) is a self-report questionnaire for the evaluation of disability and health-related quality of life after stroke.^17^ Three sections that are directly or indirectly related to upper limb recovery will be assessed: hand function, participation and overall recovery.

#### EuroQol-5D-5L

The EuroQol-5D-5L is an instrument to assess quality of life in terms of five dimensions (Mobility, Self-Care, Usual Activities, Pain/Discomfort and Anxiety/Depression) with five response categories in each of five dimensions.^18^

#### Length of stay

The length of stay will be calculated based on the date of admission to and the date of discharge from the clinical rehabilitation programme at the rehabilitation centre. These data will be requested from the administrative departments of the rehabilitation centres.

#### Ipsilesional excitability

The change in ipsilesional excitability after 10 sessions of cTBS will be assessed by measuring the RMT of the ipsilesional M1 before the first and after the final cTBS session in a subset of patients.

#### Contralesional excitability

The RMT of the contralesional M1 will be measured before the first, sixth and tenth cTBS session to assess the change in contralesional excitability during the treatment course.^19^

#### Contralesional inhibition

Contralesional inhibition will be assessed by calculating the change in average MEP amplitude of 10 TMS pulses delivered to the contralesional M1 at 110% of the RMT before and after cTBS session 1, 6 and 10 in a subset of patients.

#### Cost-effectiveness

We will perform a cost-utility analysis from a societal perspective comparing active cTBS to sham cTBS in which the effects over time will be extrapolated to investigate long-term cost-effectiveness.

Quality adjusted life years (QALYs) will be calculated from EuroQol-5D-5L outcomes and mortality using an area-under-the-curve approach based on Dutch tariffs. Quality of life will be assessed from the EuroQol-5D-5L outcomes at 90 days and 6- and 12-months post-stroke.

Costs of the interventional procedure will be calculated through a bottom-up approach estimation based on financial information from the rehabilitation centres. Other healthcare resource use will be assessed in two ways. Direct healthcare costs during clinical rehabilitation will be estimated for each centre based on the length of stay of a patient. Information on healthcare costs after clinical rehabilitation will be collected through online administration of an adaptation of the iMTA Medical Consumption Questionnaire (iMCQ) at 6- and 12-months post stroke. The iMCQ will be used to measure all direct medical costs (consultations, hospitalisations), and direct non-medical costs (family care). Productivity losses for individual patients will be collected using online administration of an adaptation of the Productivity Cost Questionnaire (iPCQ) at 6- and 12-months post-stroke. The iPCQ adaptation consists of two modules: lost productivity at paid work due to absenteeism and lost productivity at paid work due to presenteeism. Missing data will be imputed with multiple imputation and the uncertainty of the economic evaluation will be assessed with bootstrapping techniques. Cost utility and budget impact analysis plans were developed before the first patient was included (Supplemental Materials).

### Data and safety monitoring

An independent Data and Safety Monitoring Board (DSMB) will monitor the safety of participants in the trial. The DSMB will meet annually. An independent statistician will perform unblinded interim analyses on safety after 150 and 300 patients have completed the 90-days follow-up and report these directly to the DSMB. Feedback, blind to treatment, will be provided in written conclusions to the B-STARS2 project leaders. The DSMB will not perform efficacy analyses.

### Sample size calculation

Based on a two-tailed two-sample t-test power calculation with a minimal detectable effect of 6.6 points on the FM-UE scale (10% of the maximum FM-UE score), in line with recommendations from literature,^12^ a mean score at 90 days of 42 with a standard deviation of 23 (calculated from the B-STARS trial data),^20^ a statistical significance level of 0.05 and a statistical power level of 0.85, a sample size of 440 participants is required (G*Power 3).^21^ We will include 454 patients, to compensate for a loss to follow-up of 3% (based on the B-STARS trial).^20^

### Statistical analysis

#### Primary outcome analysis

The primary outcome will be the FM-UE score at 90 days post-stroke. The primary analysis will be performed using a linear mixed model in the intention-to-treat population (ITT1), defined as all randomised subjects who received at least one cTBS session. Missing data are assumed to be missing at random and a FM-UE score of 0 will be assigned in case of death. The model will include the FM-UE score at 90 days post-stroke as outcome and fixed effects for baseline FM-UE score, stratification factor 1 (ability or no ability to extend one or more fingers), age, gender, stroke subtype (ischaemic stroke or intracerebral haemorrhage) and treatment (sham cTBS or active cTBS) and a random effect for stratification factor 2 (rehabilitation centre).

#### Sensitivity analysis

A sensitivity analysis will be performed in the per-protocol population, defined as those in whom a valid FM-UE score could be assessed at 90 days post-stroke and who completed at least eight cTBS sessions. A second sensitivity analysis will be performed on complete cases, without imputation of missing data. A third sensitivity analysis will be performed based on an intention-to-treat population, consisting of all randomised subjects (ITT2).

#### Secondary outcome analysis

The secondary outcomes FM-UE, ARAT, NHPT, mRS, SIS-hand and SIS-participation and EuroQol-5D-5L at 90 days and 12 months post-stroke will be assessed using a mixed model for repeated measures or a cumulative link mixed model, depending on the type of outcome measure. The model includes fixed effects for the corresponding baseline scores, stratification factor 1, age, gender, stroke subtype (ischaemic stroke or intracerebral haemorrhage), visit (90 days or 12 months) and the interaction of treatment (sham cTBS or active cTBS) by visit and a random effect for stratification factor 2. Ipsilesional excitability will be analysed using a linear mixed model. The outcome variable is the ipsilesional RMT after the final cTBS session. The model includes fixed effects for baseline ipsilesional RMT, stratification factor 1 and treatment (sham cTBS or active cTBS) and a random effect for stratification factor 2. The length of stay will be analysed using a two-sample independent t-test. Contralesional excitability before the first, sixth and tenth cTBS session will be analysed using a mixed model for repeated measures. The outcome variable is the contralesional RMT and the model includes fixed effects for baseline contralesional RMT, cTBS session number (6 or 10) and type of treatment (sham cTBS or active cTBS) and random effects for rehabilitation centre. Contralesional inhibition after the first, sixth and tenth cTBS session will be analysed using a mixed model for repeated measures. The outcome variable is the average contralesional MEP amplitude after cTBS. The model includes fixed effects for the baseline contralesional MEP amplitude, cTBS session (1, 6 or 10) and treatment (sham cTBS; active cTBS) and a random effect for rehabilitation centre.

A statistical analysis plan was developed before the first patient was included (Supplemental Materials).

#### Interim analysis

An independent statistician will perform unblinded interim analyses for safety after 150 and 300 patients have completed the 90 days post-stroke follow-up.

### Study organisation

The B-STARS2 trial is an investigator-initiated clinical trial. The clinical trial is managed by the University Medical Center Utrecht, Utrecht, The Netherlands and monitoring is performed by Julius Clinical, Zeist, The Netherlands.

### Trial status

The first participant has been included in the trial in December 2024.

## Discussion

This trial will be the first phase 3 multicentre RCT to establish whether a paradigm of inhibitory rTMS of the contralesional M1 combined with regular care upper limb training and started within the early subacute phase after stroke, is therapeutically effective and cost-effective in promoting upper limb recovery after stroke. We will assess upper limb recovery across the functions, activities and participation domains of the International Classification of Functioning, Disability and Health up to 1-year post-stroke, providing a coherent overview of short-term and long-term effects of cTBS treatment on upper limb recovery, disability and quality of life.^22^ This trial will provide conclusive evidence on whether cTBS treatment leads to a clinically meaningful additional recovery of the upper limb after stroke, which will be decisive in the evaluation to approve it as standard of care by the National Health Care Institute in The Netherlands.

This trial will be unique in that it simultaneously investigates the proposed therapeutic mode of action. We hypothesise that contralesional cTBS inhibits contralesional M1 excitability directly after each treatment session, promoting the effect of subsequent upper limb physical therapy. We expect that suppression of contralesional excitability after individual cTBS sessions will associate with an increase in ipsilesional excitability after 10 cTBS sessions, indicative of restoration of an interhemispheric imbalance. As upper limb recovery after stroke correlates with increased excitability of ipsilesional M1,^23^ we also expect that the change in FM-UE score at 90 days post-stroke is related to the change in ipsilesional excitability after cTBS treatment.

## Supplemental Material

sj-pdf-1-eso-10.1177_23969873251355937 – Supplemental material for B-STARS2: Early contralesional continuous theta burst stimulation (cTBS) to promote upper limb recovery after stroke – Rationale and design of a phase-3 multicentre, randomised, sham-controlled, clinical trial Supplemental material, sj-pdf-1-eso-10.1177_23969873251355937 for B-STARS2: Early contralesional continuous theta burst stimulation (cTBS) to promote upper limb recovery after stroke – Rationale and design of a phase-3 multicentre, randomised, sham-controlled, clinical trial by Jord JT Vink, Tessa A Verhoeff, Willem M Otte, Miriam P van der Meulen, H Bart van der Worp, Johanna MA Visser-Meily and Rick M Dijkhuizen in European Stroke Journal
