# Supplementary material for: B-STARS2: Early contralesional continuous theta burst stimulation (cTBS) to promote upper limb recovery after stroke – Rationale and design of a phase-3 multicentre, randomised, sham-controlled, clinical trial
Source: Eur Stroke J. 2025 Jul 8:23969873251355937. Online ahead of print. doi: 10.1177/23969873251355937 (PMC12237984; doi:10.1177/23969873251355937)
Supplement: sj-pdf-1-eso-10.1177_23969873251355937 – Supplemental material for B-STARS2: Early contralesional continuous theta burst stimulation (cTBS) to promote upper limb recovery after stroke – Rationale and design of a phase-3 multicentre, randomised, sham-controlled, clinical trial [file sj-pdf-1-eso-10.1177_23969873251355937.pdf]

PROTOCOL

B-STARS 2: Brain Stimulation for Arm Recovery after Stroke 2. A phase III, randomized, double-blind, sham-controlled, clinical trial.

B-STARS 2: Brain STimulation for Arm Recovery after Stroke 2. A phase III, randomized, double-blind, sham-controlled, clinical trial.

**Protocol ID**	**NL85511.041.24**
**Short title**	**B-STARS2**
**EudraCT number**	**NA**
**Version**	**7.0**
**Date**	**08-01-2025**
**Coordinating investigator**	***Jord* JT *Vink, PhD***<br>Center for Image Sciences, UMC Utrecht & Utrecht University<br>J.j.vink-5@umcutrecht.nl
**Project leaders**	***Prof. Rick M. Dijkhuizen, PhD***<br>Center for Image Sciences, UMC Utrecht & Utrecht University<br>r.m.dijkhuizen@umcutrecht.nl<br>***Prof. H. Bart van der Worp, MD, PhD***<br>Dept. of Neurology and Neurosurgery, UMC Utrecht<br>h.b.vanderworp@umcutrecht.nl
**Principal investigator**	***Prof. Johanna M.A. Visser-Meily,* MD, PhD**<br>Dept. of Rehabilitation, Physiotherapy science and Sport, UMC Utrecht & Utrecht University<br>J.M.A.Visser-Meilij@umcutrecht.nl
**Study sites (investigator names and contact data can be found in Appendix A)**	***De Hoogstraat, Utrecht***<br>***Basalt, Den Haag***<br>***Basalt, Leiden***<br>***Adelante, Hoensbroek***<br>***MRC Aardenburg, Doorn***<br>***Heliomare, Wijk aan Zee***<br>***Vogellanden, Zwolle***<br>***Reade, Amsterdam***

	Merem, Hilversum<br>***Tolbrug, Den Bosch***<br>***Revalidatie Friesland, Beetsterzwaag***<br>***Libra Leijpark, Tilburg***<br>***Libra Blixembosch, Eindhoven***<br>***Sint Maartenskliek, Nijmegen***<br>***Revant, Breda***<br>***UMCG, Groningen***
**Sponsor (in Dutch: verrichter/opdrachtgever)**	***University Medical Center Utrecht, Utrecht***
**Subsidizing party**	***Zorginstituut Nederland***
**Independent expert (s)**	**Vera Schepers, MD**<br>Dept. of Rehabilitation, Physiotherapy science and Sport, UMC Utrecht & Utrecht University<br>v.p.m.schepers-3@umcutrecht.nl

PROTOCOL SIGNATURE SHEET

Name	Signature	Date
**Sponsor or legal representative:**<br>*Prof. Dr. J.M.A. Visser-Meily*		
**Principal investigator:**<br>*Prof. Dr. J.M.A. Visser-Meily*		

TABLE OF CONTENTS

1. INTRODUCTION AND RATIONALE 9
2. OBJECTIVES 10
3. STUDY DESIGN 10
4. STUDY POPULATION 12
4.1 Population (base) 12
4.2 Inclusion criteria 12
4.3 Exclusion criteria 12
4.4 Sample size calculation 13
5. TREATMENT OF SUBJECTS 14
5.1 Investigational treatment 14
5.2 Use of co-intervention (if applicable) 14
5.3 Escape medication (if applicable) 15
6. INVESTIGATIONAL PRODUCT 15
6.1 Name and description of investigational product(s) 15
6.2 Summary of findings from non-clinical studies 15
6.3 Summary of findings from clinical studies 15
6.4 Summary of known and potential risks and benefits 16
6.5 Description and justification of route of administration and dosage 16
6.6 Dosages, dosage modifications and method of administration 16
6.7 Preparation and labelling of Investigational Medicinal Product 16
6.8 Drug accountability 16
7. NON-INVESTIGATIONAL PRODUCT 16
7.1 Name and description of non-investigational product(s) 17
7.2 Summary of findings from non-clinical studies 17
7.3 Summary of findings from clinical studies 17
7.4 Summary of known and potential risks and benefits 17
7.5 Description and justification of route of administration and dosage 17
7.6 Dosages, dosage modifications and method of administration 17
7.7 Preparation and labelling of Non Investigational Medicinal Product 17
7.8 Drug accountability 17
8. METHODS 17
8.1 Study parameters/endpoints 17
8.1.1 Main study parameter/endpoint 17
8.1.2 Secondary study parameters/endpoints 17
8.1.3 Other study parameters (if applicable) 19
8.2 Randomization, blinding and treatment allocation 20
8.3 Study procedures 20
8.4 Withdrawal of individual subjects 23
8.4.1 Specific criteria for withdrawal (if applicable) 23
8.5 Replacement of individual subjects after withdrawal 23
8.6 Follow-up of subjects withdrawn from treatment 23

8.7 Premature termination of the study 23
9. SAFETY REPORTING 24
9.1 Temporary halt for reasons of subject safety 24
9.2 AEs, SAEs and SUSARs 24
9.2.1 Adverse events (AEs) 24
9.2.2 Serious adverse events (SAEs) 24
9.2.3 Serious Adverse Device Effects (SADEs) 25
9.2.4 Suspected unexpected serious adverse reactions (SUSARs) 26
9.3 Annual safety report 26
9.4 Follow-up of adverse events 26
9.5 Data Safety Monitoring Board (DSMB) 26
10. STATISTICAL ANALYSIS 26
10.1 Statistical analysis plan 26
10.2 Primary study parameter(s) 27
10.3 Secondary study parameter(s) 27
10.4 Other study parameters 28
10.5 Interim analysis 28
11. ETHICAL CONSIDERATIONS 28
11.1 Regulation statement 28
11.2 Recruitment and consent 29
11.3 Objection by minors or incapacitated subjects (if applicable) 29
11.4 Benefits and risks assessment, group relatedness 29
11.5 Compensation for injury 30
11.6 Incentives (if applicable) 30
12. ADMINISTRATIVE ASPECTS, MONITORING AND PUBLICATION 30
12.1 Handling and storage of data and documents 30
12.2 Monitoring and Quality Assurance 31
12.3 Amendments 31
12.4 Annual progress report 31
12.5 Temporary halt and (prematurely) end of study report 31
12.6 Public disclosure and publication policy 31
13. STRUCTURED RISK ANALYSIS 31
13.1 Potential issues of concern 31
13.2 Synthesis 34
14. REFERENCES 35

LIST OF ABBREVIATIONS AND RELEVANT DEFINITIONS

**ABR**	**General Assessment and Registration form (ABR form), the application form that is required for submission to the accredited Ethics Committee; in Dutch: Algemeen Beoordelings- en Registratieformulier (ABR-formulier)**
**AE**	**Adverse Event**
**AR**	**Adverse Reaction**
**CA**	**Competent Authority**
**CCMO**	**Central Committee on Research Involving Human Subjects; in Dutch: Centrale Commissie Mensgebonden Onderzoek**
**CV**	**Curriculum Vitae**
**DSMB**	**Data Safety Monitoring Board**
**EU**	**European Union**
**EudraCT**	**European drug regulatory affairs Clinical Tirials**
**GCP**	**Good Clinical Practice**
**GDPR**	**General Data Protection Regulation; in Dutch: Algemene Verordening Gegevensbescherming (AVG)**
**IB**	**Investigator's Brochure**
**IC**	**Informed Consent**
**IMP**	**Investigational Medicinal Product**
**IMPD**	**Investigational Medicinal Product Dossier**
**METC**	**Medical research ethics committee (MREC); in Dutch: medisch-ethische toetsingscommissie (METC)**
**(S)AE**	**(Serious) Adverse Event**
**SPC**	**Summary of Product Characteristics; in Dutch: officiële productinformatie IB1-tekst**
**Sponsor**	**The sponsor is the party that commissions the organisation or performance of the research, for example a pharmaceutical company, academic hospital, scientific organisation or investigator. A party that provides funding for a study but does not commission it is not regarded as the sponsor, but referred to as a subsidising party.**
**SUSAR**	**Suspected Unexpected Serious Adverse Reaction**
**UAVG**	**Dutch Act on Implementation of the General Data Protection Regulation; in Dutch: Uitvoeringswet AVG**
**WMO**	**Medical Research Involving Human Subjects Act; in Dutch: Wet Medisch-wetenschappelijk Onderzoek met Mensen**

SUMMARY

Rationale: Every year, about 40,000 people in the Netherlands have a stroke. After the initial admission to the hospital, about 15% of stroke survivors is admitted to a rehabilitation center because of remaining disabilities. Three out of four of these patients have upper limb dysfunction, hampering activities of daily living. Upper limb function plays a critical role in the performance of most daily life activities. In our phase II trial B-STARS, continuous theta burst stimulation (cTBS) treatment, a form of repetitive transcranial magnetic stimulation (rTMS), led to an absolute additional recovery of upper limb function of 17%, as measured with the Action Research Arm Test (ARAT) score three months after stroke. This improvement exceeds the minimal clinically important difference of 10%. cTBS treatment also resulted in a significant improvement in measures of activities and participation (of similar magnitude) and a reduction in the mean length of stay at the rehabilitation center by 18 days.

Objective: To assess the effectiveness and cost effectiveness of cTBS treatment in promoting upper limb recovery after stroke in patients admitted to a rehabilitation center.

Study design: A phase III, multi-center, double-blind, randomized, sham-controlled, clinical trial.

Study population: 454 patients aged 18 years or older with a first-ever ischemic stroke or intracerebral hemorrhage and a unilateral arm paresis, defined by a Motricity Index between 9 and 99, in whom cTBS treatment can be started within 3 weeks after stroke onset.

Intervention: 10 daily sessions of cTBS delivered over the contralesional primary motor cortex during a period of 2 weeks, immediately before regular care physical therapy of the affected upper limb.

Main study parameters/endpoints: The primary endpoint will be the score on the upper extremity section of the Fugl-Meyer assessment (FM-UE) at 90 days after stroke. Secondary endpoints will include the score on the FM-UE at one year and the scores on the Action Research Arm Test, Nine Hole Peg Test, Stroke Impact Scale, EuroQol 5 Dimensions and modified Rankin Scale at 90 days and one year after stroke

Nature and extent of the burden and risks associated with participation, benefit and group relatedness: Data from our phase II trial B-STARS indicate that cTBS treatment results in 17% additional recovery of upper limb function, as measured with the ARAT at three months after stroke. This additional recovery led to a significant improvement in the activity and participation domain (of similar magnitude) and a reduction in the length of stay at the rehabilitation center by 18 days on average. Risks associated with active cTBS were limited to headache (<4%) and muscle pain (<1%). Analysis of interviews with a selected number of patients showed that cTBS was well tolerated.

1. INTRODUCTION AND RATIONALE

Every year about 40,000 people in the Netherlands have a stroke. Despite advances in the treatment of stroke in the acute phase, most patients experience lasting disability, including persistent impairments in upper limb function, hampering activities of daily living, societal participation and quality of life.[1] There is no proven effective treatment for these persistent impairments. In recent years, non-invasive brain stimulation techniques, such as repetitive transcranial magnetic stimulation (rTMS), have shown promise as a therapeutic means to promote functional recovery after stroke.[2]

In 2022, our team completed a phase II, single-blinded, mono-center randomized controlled trial of rTMS in sixty stroke patients at rehabilitation center De Hoogstraat.[3] In this trial a specific rTMS protocol, involving continuous theta-burst stimulation (cTBS), started within three weeks after stroke, improved upper limb function up to at least one year after stroke.[3] The treatment was safe and well-tolerated and was associated with a reduction in the duration of admission to the rehabilitation center by a mean of 18 days. These findings corroborate results from systematic reviews and meta-analyses of published studies, suggesting benefit of rTMS on upper limb function if started within the first month after stroke.[2,4,5] Because our trial was a relatively small (n = 60) single-center study and the methods and results of the trials included in abovementioned meta-analyses were heterogeneous, these findings are insufficient to change clinical practice. In a recent module of the Dutch guideline ‘Ischemic stroke and intracerebral hemorrhage’, it is concluded that inhibitory rTMS in the first weeks after stroke is a promising treatment for improvement of upper limb function, but that this needs to be tested in high-quality phase III trials before rTMS can be recommended as a standard of care.

We therefore propose a multicenter, randomized, controlled, phase III clinical trial of cTBS for upper limb recovery after stroke across multiple rehabilitation centers in the Netherlands. Therapeutic efficacy will be measured with the Fugl-Meyer Upper Extremity test at 90 days and one-year post-stroke. Additionally, cost effectiveness will be assessed by a healthcare technology assessment expert. If the treatment is proven effective, implementing rTMS in rehabilitation therapy can improve functional recovery in thousands of stroke patients in the Netherlands annually. Moreover, a proven effective treatment would likely result in significant cost savings due to shorter duration of admission to a rehabilitation center and faster societal integration.

2. OBJECTIVES

Primary Objective:

- To assess whether 10 sessions of cTBS of the contralesional primary motor cortex (M1) combined with regular care upper limb training, started within three weeks after stroke onset and continued every working day for two weeks, reduces upper limb impairment at 90 days after stroke, compared to sham stimulation.

Secondary Objective(s):

To assess whether 10 sessions of cTBS of the contralesional M1 combined with regular care upper limb training, started within three weeks after stroke onset and continued every working day for two weeks:

- improve upper limb activity, manual dexterity and quality of life, and reduces disability and dependence at 90 days and 12 months after stroke, compared to sham stimulation;
- reduce upper limb impairment at 12 months after stroke, compared to sham stimulation;
- are cost-effective compared to sham stimulation;
- reduce excitability of the contralesional M1 during the 2-week treatment period, compared to sham stimulation;
- increase ipsilesional M1 excitability after 10 treatment sessions, compared to sham stimulation.

3. STUDY DESIGN

We will perform a multi-center, double-blind, randomized, sham-controlled, clinical trial. Subjects will be recruited from multiple rehabilitation centers in the Netherlands over a period of 51 months (Q2 of 2024 to Q4 of 2028). The total duration of follow-up for each participant is until one year after stroke. The total study duration is 63 months.

Baseline assessment will be performed before the start of the intervention, which must occur within 21 days after stroke onset. Follow-up assessments will be performed at 90 days (+/- 7 days) and 12 months (+/- 14 days) post-stroke (Figure 1). Outcome assessments are described in Table 1 and will be described in more detail in section 8.3.

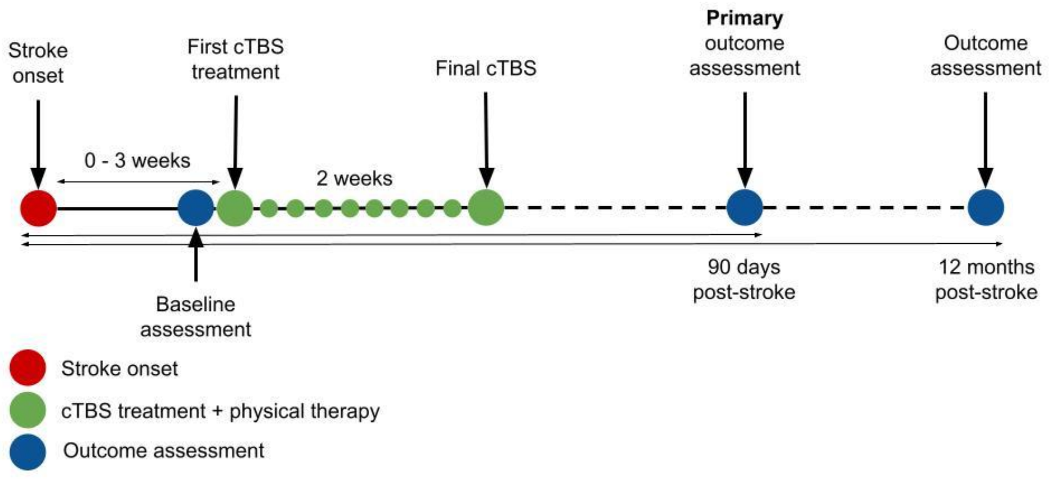

Figure 1. Study timeline.

Table 1. Outcome assessments

Assessment	Timepoint	Description	Abbreviation
Fugl-Meyer – Upper Extremity	Baseline, 90 days 12 months	Upper extremity impairment test	FM-UE
Action Research Arm Test	Baseline, 90 days 12 months	Upper extremity activity test	ARAT
Nine Hole Peg Test	Baseline, 90 days 12 months	Manual dexterity test	NHPT
Modified Rankin Scale	Baseline, 90 days 12 months	Disability and dependence questionnaire	mRS
Barthel Index	Baseline, 90 days 12 months	Activities of daily living questionnaire	BI
Stroke Impact Scale – Hand	Baseline, 90 days 12 months	Hand function questionnaire	SIS-hand
Stroke Impact Scale - participation	Baseline, 90 days 12 months	Participation questionnaire	SIS-par
EuroQol - 5 dimensions	Baseline, 90 days, 6 months,12 months	Quality of life questionnaire	EQ-5D-5L
Length of stay		Length of stay in the rehabilitation center	LOS

Contralesional excitability	Before the first, 6th and 10th cTBS session	Resting motor threshold of the contralesional M1	cRMT
Ipsilesional excitability	Before the 1st cTBS session and after the 10th cTBS session.	Resting motor threshold of the ipsilesional M1	iRMT
Contralesional inhibition	Before and after the 1st, 6th and 10th cTBS session	Ratio between the average motor-evoked potential amplitude before and after cTBS.	CI
TMS disruption	Baseline	Change in tapping frequency of the affected hand during disruption of contralesional M1 activity	

4. STUDY POPULATION

4.1 Population (base)

Every year, some 3,000 stroke patients are admitted to clinical rehabilitation programs across rehabilitation centers in the Netherlands (Vektis declaratieset MSR 2020). 75% of whom experiences loss of upper limb function and may be eligible for inclusion in our trial. 454 of these patients will be recruited from rehabilitation centers in the Netherlands.

4.2 Inclusion criteria

To be eligible to participate in this study, a participant must meet all the following criteria:

- Age, 18 years or older
- First-ever unilateral ischemic stroke or intracerebral hemorrhage in a cerebral hemisphere or in the brainstem;
- Unilateral upper limb paresis with a motricity index between 9 and 99;
- Possibility to start cTBS treatment within 21 days after stroke onset;
- Signed informed consent.

4.3 Exclusion criteria

A potential subject who meets any of the following criteria will be excluded from participation in this study:

- Upper limb paresis prior to stroke onset;

- Absolute contra-indication to TMS
  - Magnetically sensitive objects implanted in or guided through the head or neck area (e.g. cochlear implants, implanted neurostimulator, pacemaker or defibrillator leads, metal splinters, metal fragments or metal clips) with the exception of dental work (e.g. fillings, braces or implants);
  - History of epilepsy;
  - Pregnancy;
  - Other contra-indications that may potentially be harmful as determined by the treating rehabilitation physician;
- Incapacity or severe impairments (i.e. extreme fatigue, major communication deficits) that can impede study participation as determined by the treating rehabilitation physician;
- Life expectancy shorter than one year.

4.4 Sample size calculation

The required sample size to test our hypothesis (superiority) in two groups is 440 subjects, based on a two-sided two-sample t-test power calculation with a minimal detectable effect of 6.6 points on the FM-UE scale (10% of the maximum score of 66, a mean score at 90 days of 42 and a standard deviation of 23 (calculated from the B-STARS trial data), a statistical significance level of 0.05 and statistical power of 0.85 (G*power 3).[6]

A study that specifically examined clinically important differences of FM-UE scores in stroke patients found that these ranged from 4.25 to 7.25 points, depending on the different facets of UE movement.[7] A minimal clinically important difference of 6.6 points (10% of the maximum score FM-UE score) is in line with the module ‘Noninvasive brain stimulation with rTMS’ for the Dutch guideline ‘Ischemic stroke and intracerebral hemorrhage’.[8]

To compensate for possible loss to follow-up, we will include 454 patients. This sample size is also sufficient to demonstrate a treatment effect on secondary outcome measures. For example, for the ARAT, which tests upper limb-specific activity, a sample size of at least 396 would be required, based on a minimal clinically important difference of 5.7 and a mean score and standard deviation of 30 and 23, respectively (calculated from the B-STARS trial data), a statistical significance level of 0.05, and a statistical power of 0.8 (gigacalculator.com).

5. TREATMENT OF SUBJECTS

5.1 Investigational treatment

Active cTBS

The intervention consists of 10 daily sessions of active or sham cTBS, delivered over the contralesional primary motor cortex (M1) in a period of 10 consecutive working days within a period of 17 days. The first cTBS session takes place within 21 days after stroke onset. cTBS treatment will be delivered prior to at least 40 minutes of regular care upper limb therapy, with a maximum time interval between cTBS and upper limb therapy of 15 minutes. Regular care upper limb therapy will be performed according to local protocols, which are based on national guidelines from the royal Dutch Society for Physical Therapy, and will be adjusted based on the patient's level of upper limb impairment. TMS procedures will be performed in accordance with recent safety guidelines for TMS.[2]

Stimulation location is defined as the position on the scalp at which motor evoked potentials (MEPs) with the largest peak-to-peak amplitude could be evoked in the first dorsal interosseous (FDI) muscle of the unaffected arm by delivering TMS pulses and monitoring the electromyogram (EMG). Stimulation intensity is set at 70% of the resting motor threshold (RMT), which is defined as the minimum machine output at which stimulation evokes at least 5 of 10 MEPs with a peak-to-peak amplitude of over 50 µV with the TMS coil oriented 45 degrees to the midline and an interstimulus interval of 7s. The stimulation location and intensity are determined before the 1st, 6th and 10th cTBS session.

cTBS consists of continuous delivery of bursts of 3 biphasic stimuli at 50 Hz repeated at 5 bursts per second for a duration of 40 seconds resulting in a total of 600 stimuli. During treatment, the TMS coil is placed on the scalp and oriented at 45 degrees to the midline.

Active cTBS will be delivered using an active figure-of-eight TMS coil.

Sham cTBS

Sham procedures are identical to the active cTBS procedures, except that sham cTBS will be delivered using a sham figure-of-eight coil. The sham coil looks and sounds identical to the active coil, but the internal wiring of the sham coil is such that the active electrical field that is induced by the coil is highly reduced, so that the effective electrical field does not reach the cortical layer.

5.2 Use of co-intervention (if applicable)

Not applicable.

5.3 Escape medication (if applicable)
Not applicable.

6. INVESTIGATIONAL PRODUCT

6.1 Name and description of investigational product(s)
CTBS treatment will be delivered using a therapeutic biphasic magnetic stimulator that supports the continuous theta burst stimulation protocol described in section 5.1 with a pulse width between 250 and 330 microseconds, includes cooling and an integrated one- or multi-channel electromyography (EMG) system and three separate figure-of-eight TMS coils for motor-threshold determination, active and sham stimulation. Currently, the following three CE-certified TMS devices with an intended use that covers the application described in this protocol are available:

- The MagVenture MagPro R30 stimulator with an MCF-B70 figure-of-eight TMS coil;
- The Deymed DuoMag XT-100 stimulator with a figure-of-eight TMS coil.
- The Neurosoft neuro-MSX stimulator with angulated figure-of-eight TMS coil.
- The MagStim Super Rapid[2] stimulator with cooled Air Film Coil.
- The MagStim Horizon 3.0 with figure-of-eight TMS coil.

TMS coil placement during cTBS treatment will be guided and recorded using a compatible MRI-guided neuronavigation system that supports standard head model import. The following three compatible CE-certified neuronavigation systems with an intended use that covers the application described in this protocol are available:

- The Brain Science Tools neural navigator: Essentials and Atlas variants. Both variants will be used in combination with the headband of the Atlas variant.
- The Localite TMS navigator
- MagStim StimGuide+ (as part of the Horizon 3.0 system)

6.2 Summary of findings from non-clinical studies
A recent meta-analysis of preclinical studies on the effects of rTMS on motor function showed that 89% (8 out of 9) of studies found a significant effect on motor recovery based on 21 different tests performed at various time points, and 67% (4 out of 6) of studies reported a reduction in infarct size.[9]

6.3 Summary of findings from clinical studies
We performed a phase II, single-blinded, mono-center, randomized controlled trial of rTMS in sixty stroke patients at rehabilitation center De Hoogstraat (NL58423.016.41).[3] In this trial, we showed that cTBS treatment improves upper limb function up to at least one year after stroke. The treatment was safe and well-tolerated and was associated with a reduction in the duration of admission to the rehabilitation center by 18 days.[3] These findings corroborate results from a systematic review and meta-analyses of published

studies, revealing benefit of rTMS on upper limb function if started within the first month after stroke.[2,4,5]

6.4 Summary of known and potential risks and benefits

Data from our phase II trial (B-STARS) shows that cTBS treatment leads to a clinically relevant improvement in upper limb recovery, with 17% (n 60; 9.6 points; 95%CI 1.2–17.9) additional recovery of upper limb activity, as measured with the ARAT score three months after stroke.[3] This additional recovery led to a significant improvement in the activity and participation domain (of similar magnitude) of the International Classification of Functioning, Disability and Health (ICF) model, and a reduction in the length of stay at the rehabilitation center by 18 days on average.[3] These findings corroborate results from a systematic review and meta-analysis of published studies, revealing benefit of rTMS on upper limb function if started within the first month after stroke. [2,4,5]

The most serious adverse effect is the occurrence of a seizure at a crude risk of approximately 0.02% per stimulation session, as reported in a systematic review of 67 studies with 1,040 subjects and >4,500 sessions.[10] More common adverse events during and after TBS are transient headache and neck pain, reported by <3% of subjects.[10] CTBS treatment did not lead to seizures in our phase II trial.[3] We only observed mild side effects, such as headache (<4%) and muscle pain (<1%) and the intervention was reported to be tolerable and comfortable.[3]

6.5 Description and justification of route of administration and dosage

The dose (70% of RMT) and the route of administration (transcranial magnetic stimulation) is based on our phase II B-STARS trial.[3]

6.6 Dosages, dosage modifications and method of administration

See previous response.

6.7 Preparation and labelling of Investigational Medicinal Product

Not applicable

6.8 Drug accountability

Not applicable.

7. NON-INVESTIGATIONAL PRODUCT

Not applicable.

7.1 Name and description of non-investigational product(s)

7.2 Summary of findings from non-clinical studies

7.3 Summary of findings from clinical studies

7.4 Summary of known and potential risks and benefits

7.5 Description and justification of route of administration and dosage

7.6 Dosages, dosage modifications and method of administration

7.7 Preparation and labelling of Non Investigational Medicinal Product

7.8 Drug accountability

8. METHODS

8.1 Study parameters/endpoints

8.1.1 Main study parameter/endpoint

The primary endpoint is the upper extremity section of the Fugl-Meyer assessment (FM-UE) at 90 days post-stroke. The FM-UE assesses upper limb motor impairment on a scale from 0 to 66 points, with a higher score indicating less impairment. The FM-UE will be assessed by certified assessors. Before certification, assessors will receive on-site training by the team of coordinating investigators and must successfully complete an FM-UE exam. See section 8.3.

8.1.2 Secondary study parameters/endpoints

A detailed description of secondary outcomes and assessments are provided in section 8.3.

Secondary endpoints are the FM-UE at 12 months post-stroke and the Action Research Arm Test (ARAT), nine hole peg test (NHPT), modified Rankin scale (mRS), hand and participation sections of the stroke impact scale (SIS), Barthel Index and the EuroQol - 5 dimensions – 5 levels (EQ-5D-5L) at 90 days and 12 months post-stroke.

An additional secondary outcome is the change in contralesional corticospinal excitability during the course of treatment. Therefore, the contralesional RMT is measured prior to each cTBS session (these data are already acquired to determine stimulation intensity). Contralesional inhibition is assessed to determine whether cTBS treatment had a direct inhibitory effect. This is done for cTBS session 1, 6 and 10 by acquiring the average MEP amplitude for 10 TMS pulses delivered to the contralesional M1 at 110%RMT.

Furthermore, a secondary outcome is the ipsilesional corticospinal excitability directly after the final cTBS session. Therefore, the ipsilesional RMT is measured before the first and after the final cTBS session.

TMS disruption will be assessed at baseline to determine whether contralesional M1 activity is maladaptive for affected hand performance, as this may predict cTBS treatment outcome. This will only be performed at De Hoogstraat Rehabilitation.

Cost-effectiveness

We will perform a cost-utility analysis alongside the trial from a societal perspective with a time horizon of one year comparing active cTBS to sham cTBS. In a scenario analysis, we will show the impact on cost-effectiveness, if effects at one year would be extrapolated over time. Sham stimulation could have a placebo effect; however, the magnitude of the placebo effect is unsure. A scenario analysis will be performed to show the potential impact of the placebo effect on cost-effectiveness results.

Quality of life will be assessed by combining mortality and quality of life using an area-under-the-curve approach. Quality of life will be assessed with the EQ-5D-5L. The Dutch tariffs will be used to calculate answer into a utility score. This utility score can range up to 1, in which 0 corresponds to death and 1 corresponds to perfect health.

Costs of the interventional procedures itself will be calculated with a bottom-up approach or estimated based on financial information from the rehabilitation centers. In a scenario analysis, we will estimate cost-effectiveness if we exclude navigation costs during the procedure.

Other healthcare resource use will be assessed in two ways. Direct healthcare costs during clinical rehabilitation will be estimated for each center based on the length of stay (LOS) of a patient. LOS will be calculated based on the date of admission and the date of discharge at the rehabilitation center. These dates will be requested from the administrative department of each rehabilitation center. Healthcare costs after clinical rehabilitation will be collected through CASTOR at 6 and 12 months post-stroke using an adaptation of the iMTA Medical Consumption Questionnaire (iMCQ). Data will be extrapolated to fill in missing data caused by the recall-period of three months. The iMCQ will be used to measure all direct medical costs (consultations, hospitalizations), and direct non-medical costs (family care). Healthcare resource use will be multiplied with Dutch unit costs to determine healthcare costs. Unit costs will be used as described in the Dutch Costing guideline; NZA tariffs or internal UMCU tariffs if both are missing.

The Productivity Cost Questionnaire (iPCQ) will be sent out through CASTOR at 6 and 12 months post-stroke to collect productivity losses for each individual patient. The iPCQ will consist of two modules: lost productivity at paid work due to absenteeism and lost productivity at paid work due to presenteeism. Data will be extrapolated to fill in missing data caused by the recall period of four weeks.

Missing data will be imputed with multiple imputation and the uncertainty of the economic evaluation will be assessed with bootstrapping techniques.

To be able to extrapolate trial findings and to perform other scenario analysis; a decision-analytical model (a decision tree) will be developed. In this decision tree, we will correlate trial findings in EQ-5D-5L and costs to outcomes in Fugl-Meyer.

A detailed protocol for the cost-effectiveness study will be prepared separately.

8.1.3 Other study parameters (if applicable)

Baseline values

Demographic information

Sex, age, education level based on the international standard classification of education (ISCED), handedness, marital status, work status, ethnicity.

Medical history

Diabetes mellitus, hypertension, smoking

Medication

Current medication

Stroke parameters

Type of stroke, stroke location (including affected hemisphere or brain stem), National Institutes of Health Stroke Scale (NIHSS) at admission to the hospital, date of stroke onset, treatment with intravenous thrombolysis or endovascular thrombectomy in the acute phase, Montreal Cognitive Assessment (MOCA) at admission to rehabilitation center, O-letter cancellation test, pre-stroke score on the mRS.

Baseline neurological and functional status

FM-UE, ARAT, NHPT, mRS, SIS, BI, EQ-5D-5L, ability to extend 1 or more fingers of the paretic arm, motricity index,

Baseline electrophysiology

Corticospinal excitability

8.2 Randomization, blinding and treatment allocation

Patients will be randomly allocated to receive active or sham continuous theta burst stimulation (cTBS) treatment. Randomization will be executed through a web-based allocation service (CASTOR) that generates a unique study number for each participant. Randomization is stratified based on the ability to extend one or more fingers of the paretic arm and the rehabilitation center at which the participant is recruited. Information on treatment allocation will be kept separate from the main study database. Patients will be randomly allocated to group A or group B, to allow double-blinding.

Sham cTBS will be performed using a sham TMS coil, which looks identical to an active TMS coil but does not produce effective stimulation. The sham and active TMS coils will be labeled with a code (A/B) by the coordinating investigator who is not involved in any of the TMS treatments. The TMS therapist performs randomization and patients will be allocated to receive treatment with coil A or B, depending on their group allocation (A/B). Therefore, the TMS therapists and patients are unaware of treatment allocation. Blinding of the patient and therapist will be maintained until the last patient has completed the final follow-up (at 12 months post-stroke). Only one of the coordinating investigators can link treatment allocation to the active or sham condition. The independent trial statistician will combine data on treatment allocation with the clinical data to report to the data safety monitoring board (DSMB). The treatment allocation code will not be broken until the database has been locked, which is after the all data have been cleaned. The moment of datalock is defined in the statistical analysis plan. Cleaning of the data will be performed by a coordinating investigator who is unaware of treatment allocation.

8.3 Study procedures

A local investigator will perform screening and the informed consent procedure described in section 11.1. After written informed consent is obtained, baseline assessments will be performed. Baseline assessment duration is up to 90 minutes. Descriptions of the study procedures to determine the outcome measures (Table 1) are provided below.

After the baseline assessments, patients undergo the intervention described in section 5.1. The following data related to cTBS treatment is acquired: date and time of each treatment, the resting motor threshold, experienced side-effects and the number of minutes of upper limb therapy after the previous session. At the end of treatment course, the following data is acquired: the number of completed treatments,

the duration of the treatment course in days, and whether the patient thinks he/she received active or sham treatment.

At 90 days and 12 months post-stroke, the outcome measures described in table 1 are reassessed. Follow-up assessment duration is up to 90 minutes per follow-up. Detailed descriptions of these tests are provided below.

After the patient has been discharged, the total amount of upper limb therapy received during clinical rehabilitation and the length of stay at the rehabilitation center are acquired.

Upper extremity section of the Fugl-Meyer assessment (FM-UE)
The upper extremity section of the Fugl-Meyer assessment is a reliable and valid motor performance test consisting of 33 tasks performed with the affected upper limb. The FM-UE evaluates the ability to make movements outside the synergistic pattern. Performance on each task is rated as 0, 1 or 2, with a maximum score of 66 points and with a higher score indicating less impairment.

Action research arm test (ARAT)
The ARAT is an upper limb performance test which assesses the ability to perform gross movements and the ability to grasp, move and release objects differing in size, weight and shape. The test consists of 19 items, rated on 4-point ordinal scales (0 to 3), with a maximum score of 57. A higher score indicates better performance.

Nine Hole Peg Test (NHPT)
The NHPT examines fine motor skills and manual dexterity. The patient must put 9 pegs into slots and individually place them back into a bin as quickly as possible with the affected hand. The test's outcome is the execution duration, with a maximum duration of 50 seconds, and the number of pegs that have been moved (within the 50 second time window), with a maximum of 18 points (1 point per peg movement). Reliability and validity have been demonstrated in stroke patients.

Modified Rankin Scale (mRS)
The modified Rankin Scale measures the degree of disability and dependence on an ordinal scale from 0 (no symptoms) to 5 (severe disability) and 6 (death).

O-letter cancellation test (OCT), only at baseline

The O-letter cancellation test is a screening tool for unilateral spatial neglect. In the OCT there are 40 O-letters interspersed with other letters shown on a piece of paper. The patient must cross out all the O-letters. The time to complete the test and the number of correctly crossed out O-letters on the contralateral and ipsilesional side are recorded.

Stroke Impact Scale

The Stroke Impact Scale (SIS) is a self-report questionnaire for the evaluation of disability and health-related quality of life after stroke. Three sections that are directly or indirectly related to upper limb recovery will be assessed: hand function, participation and overall recovery.

Barthel index

The BI is an ordinal scale used to measure performance in 10 activities of daily living. Test scores range from 0 to 100, with higher scores indicating better performance in these activities

EuroQol-5D-5L

The EuroQol-5D-5L is a quality-of-life questionnaire. It consists of 5 questions with 5 answer possibilities each. Each question captures one dimension of quality of life: mobility, self-care, daily activities, pain or other complaints, and anxiety/depression.

Contralesional corticospinal excitability

Corticospinal excitability is determined based on the RMT (defined under section 5.1). The contralesional RMT is assessed before the 1st, 6th and 10th cTBS session, as part of the treatment procedure described under section 5.1. Therefore, it does not require additional measurements.

Ipsilesional corticospinal excitability (optional)

Ipsilesional excitability (i.e. the RMT) is assessed at baseline and directly after 10 cTBS sessions.

Contralesional inhibition (optional)

Contralesional inhibition is determined based on the ratio between the average MEP amplitude in response to 10 stimuli at 110% RMT before and after cTBS sessions 1, 6, and 10. The stimulation location is the motor hotspot, as identified prior to cTBS

treatment (to determine the cTBS treatment location). The RMT is also determined before the 1st, 6th and 10th cTBS treatment (to determine cTBS stimulation intensity).

TMS disruption (optional)
TMS disruption consists of a train of 30 active or sham TMS pulses at 10Hz and 90% RMT, delivered over the contralesional M1. The participant is instructed to start tapping the index finger of the affected hand at the onset of an auditory stimulus at 0s for a duration of 3 seconds at maximum frequency. The train of TMS pulses covers the entire finger tapping period. Finger tapping frequency is recorded using a key on a keyboard with the wrist fixed to the table using a velcro strap to minimize variability in task execution. 5 sessions of sham and 5 sessions of active TMS interference of the contralesional M1 are acquired.

8.4 Withdrawal of individual subjects

Patients who decide to withdraw from treatment will continue to participate in follow-up assessments, unless they withdraw from the study completely. Subjects can leave the study at any time for any reason if they wish to do so without any consequences. The investigator can decide to withdraw a subject from the study for urgent medical reasons.

8.4.1 Specific criteria for withdrawal (if applicable)

Not applicable.

8.5 Replacement of individual subjects after withdrawal

Subjects who withdraw from the study will not be replaced.

8.6 Follow-up of subjects withdrawn from treatment

Patients who withdraw from treatment can agree to participate in follow-up.

8.7 Premature termination of the study

If the Sponsor, the Investigator(s), or Regulatory Authorities discover any condition during the study that indicate that the study or study site should be terminated, this action may be taken after appropriate consultation between the Sponsor and the Investigator(s). The Sponsor has the right to terminate the participation of either an individual site or the complete study at any time. This action may be taken based on the recommendation of the independent DSMB.

Reasons for termination may include, but are not limited to, the following:

- The incidence and severity of AEs in this or other studies indicates a potential health hazard to subjects;
- Subject enrolment is unsatisfactory;

- Data recording is inaccurate or incomplete to an unacceptable extent;
- Investigator(s) do not adhere to the protocol or applicable regulatory guidelines in conducting this study;
- Submission of knowingly false information from the study site to the Sponsor or regulatory authorities;
- Results of an interim analysis supporting terminating the study.

In the event that the study is terminated early, the Sponsor will provide specific guidance to investigational sites regarding the end-of-study procedures.

9. SAFETY REPORTING

9.1 Temporary halt for reasons of subject safety

In accordance to section 10, subsection 4, of the WMO, the sponsor will suspend the study if there is sufficient ground that continuation of the study will jeopardize subject health or safety. The sponsor will notify the accredited METC without undue delay of a temporary halt including the reason for such an action. The study will be suspended pending a further positive decision by the accredited METC. The investigator will take care that all subjects are kept informed.

9.2 AEs, SAEs and SUSARs

9.2.1 Adverse events (AEs)

Adverse events are defined as any undesirable experience occurring to a subject during the study, whether or not considered related to cTBS treatment. All (serious) adverse events reported spontaneously by the subject or observed by the investigator or his staff during a 3-week period after the start of the first cTBS treatment will be recorded. Reporting is limited to a 3-week period because the side effects of rTMS are limited to 24 hours after stimulation. Additionally, stroke survivors have an increased risk of developing secondary medical conditions that are unrelated to cTBS treatment. During the follow-up period, in which only motor function tests and questionnaires are acquired AEs and SAEs will not be reported.

9.2.2 Serious adverse events (SAEs)

A serious adverse event is any untoward medical occurrence or effect that

- results in death;
- is life threatening (at the time of the event);
- requires hospitalization or prolongation of existing inpatients' hospitalization;
- results in persistent or significant disability or incapacity;

- is a congenital anomaly or birth defect; or
- any other important medical event that did not result in any of the outcomes listed above due to medical or surgical intervention but could have been based upon appropriate judgement by the investigator.

An elective hospital admission will not be considered as a serious adverse event.

The investigator will report all SAEs to the sponsor without undue delay after obtaining knowledge of the events, except for expected SAEs (see appendix B).

The sponsor will report all unexpected SAEs through the web portal *ToetsingOnline* to the accredited METC that approved the protocol, within 7 days of first knowledge for SAEs that result in death or are life threatening followed by a period of maximum of 8 days to complete the initial preliminary report. All other unexpected SAEs will be reported within 15 days after the sponsor has first knowledge of the serious adverse events.

Expected SAEs are events known to occur in patients with (subacute) stroke. Expected SAEs are defined in the protocol (Appendix B). Expected SAEs are excluded from expedited reporting but should be documented by the local investigator in the eCRF within 7 days of the investigator's first awareness about the event.

9.2.3 Serious Adverse Device Effects (SADEs)

Unexpected adverse device effects are SADEs if the following three conditions are met:
- the event must be serious (see Section 9.2.2);
- there must be a certain degree of probability that the event is a harmful and an undesirable reaction to the medical device described in section 6.1.
- the event must be unexpected, that is, the nature and severity of the event are not in agreement with the device information as reported in the IFU.

SADEs must be reported to the sponsor within 24 hours of first knowledge. The sponsor will report all SADEs through the web portal *ToetsingOnline* to the accredited METC that approved the protocol, within 7 days of first knowledge for SAEs that result in death or are life threatening followed by a period of maximum of 8 days to complete the initial preliminary report. All other unexpected SAEs will be reported within 15 days after the sponsor has first knowledge of the serious adverse events.

9.2.4 Suspected unexpected serious adverse reactions (SUSARs)

Not applicable.

9.3 Annual safety report

Not applicable.

9.4 Follow-up of adverse events

All AEs will be followed until they have abated, or until a stable situation has been reached. Depending on the event, follow up may require additional tests or medical procedures as indicated, and/or referral to the general physician or a medical specialist. SAEs need to be reported till end of study within the Netherlands, as defined in the protocol

9.5 Data Safety Monitoring Board (DSMB)

An independent Data and Safety Monitoring Board (DSMB) will oversee the safety of patients in the trial. They will work in accordance with a dedicated charter and will follow processes recommended by the DAMOCLES statement. The DSMB will meet in person or online at least annually. An independent statistician will perform unblinded interim analyses as described under 10.5. The independent statistician will report the outcome of the interim analyses directly to the DSMB, as well as listings of all SAE reports and unblinded aggregate summaries of data by treatment group for review in closed meetings. Feedback, blind to treatment, will be provided in written conclusions to the sponsor and the B-STARS2 project leaders. Should the sponsor decide not to fully implement the advice of the DSMB, the sponsor will send the advice to the reviewing METC, including a note to substantiate why (part of) the advice of the DSMB will not be followed.

10. STATISTICAL ANALYSIS

10.1 Statistical analysis plan

The analysis and reporting of the trial will be in accordance with CONSORT guidelines. Before follow-up will have been completed, a statistical analysis plan (SAP) will be developed that will specify: (i) Hypotheses to be tested; (ii) Treatment effects to be estimated in order to satisfy the primary and secondary objectives of this trial; (iii) Technical description of the statistical methodology and procedures for performing the statistical analysis of outcome measures and SAE data; (iv) Primary, secondary, and sensitivity analyses; and (v) Subgroup analyses. The final SAP will be published before the first patient is included in the trial. The SAP will be signed off by the trial Executive Committee (Appendix C) and the trial statistician

and then published. The final statistical analyses will be performed once recruitment has ceased, final follow-up has been completed, final data have been checked and any errors corrected, and the database has been locked.
All analyses will be performed according to the intention-to-treat (ITT) principle. Baseline data by treatment allocation will be reported with statistical procedures. Missing values for baseline characteristics will be reported. Missing baseline characteristics will be imputed using multiple imputation.

10.2 Primary study parameter(s)

The primary outcome is the FM-UE score at 90 days poststroke. The FM-UE reflects upper limb impairment on an ordinal scale from 0 (maximum impairment) to 66 points (no impairment). The primary analysis is performed using a linear mixed model in the intention-to-treat population, including all randomized subjects who started at least one cTBS session. Missing data are assumed to be missing at random and a FM-UE score of 0 will be assigned in case of death. The model includes at least the baseline FM-UE score; the stratification factor (ability versus no ability to extend one or more fingers), age, stroke subtype (ischemic stroke vs. intracerebral hemorrhage), and treatment (sham cTBS; active cTBS). The model includes a random effect for rehabilitation center.

<u>Sensitivity analyses</u>

Sensitivity analyses will be performed in the per-protocol population (defined as those in whom a valid FM-UE score could assessed at 90 days post-stroke and who completed at least eight cTBS sessions).

<u>Subgroup analyses</u>

Predefined subgroup analyses will be based on baseline FM-UE extremity score (FM ≤ 20 versus FM > 20); stroke subtype (ischemic stroke versus intracerebral hemorrhage); stroke location (cortical versus subcortical versus brainstem); time to start of treatment (within 14 days versus 15 to 21 days). Additional subgroup analyses may be described in the statistical analysis plan.

10.3 Secondary study parameter(s)

Secondary endpoints are the FM-UE at 12 months post-stroke and the ARAT, NHPT, mRS, SIS-hand, SIS-participation and the EQ-5D at 90 days and 12 months post-stroke. These secondary endpoints are analyzed using a mixed model repeated measures or cumulative link mixed model, depending on the nature of the outcome measure. The model includes the baseline score; the stratification factor (ability versus no ability to

extend 1 or more fingers), visit (90 days and 12 months post-stroke), and the interaction of treatment (sham cTBS; active cTBS) by visit. The model includes a random effect for rehabilitation center. Additional model parameters and predefined subgroup analyses are described in the SAP.

The length of stay will be analyzed using a two-sample independent t-test.

Ipsilesional excitability will be analyzed using a linear mixed model. The outcome variable is the ipsilesional RMT after the final cTBS session. The model includes fixed effects for the ipsilesional RMT before the first cTBS session, stratification factor (ability versus no ability to extend 1 or more fingers) and treatment (sham cTBS; active cTBS) and a random effect for rehabilitation center.

Contralesional excitability before the 1st, 6th and 10th cTBS session will be analyzed using a mixed model for repeated measures. The outcome variable is the contralesional RMT determined before the 1st, 6th and 10th cTBS session. The model includes fixed effects for baseline RMT, cTBS session number (1, 6 or 10) and type of treatment (sham cTBS; active cTBS) and a random effect for rehabilitation center.

Contralesional inhibition after the 1st, 6th and 10th cTBS session will be analyzed using a mixed model for repeated measures. The outcome variable is the contralesional MEP amplitude after cTBS. The model includes fixed effects for and the contralesional MEP amplitude, cTBS session (1, 6 or 10) and treatment (sham cTBS; active cTBS) and a random effect for rehabilitation center.

10.4 Other study parameters

Not applicable.

10.5 Interim analysis

With respect to safety, an independent statistician will perform unblinded interim analyses on reported SAE and AE after 150 and 300 patients have completed the 90 days post-stroke follow-up.

11. ETHICAL CONSIDERATIONS

11.1 Regulation statement

Study conduct will be according to the principles of the Declaration of Helsinki (64th WMA General Assembly, Fortaleza, Brazil, October 2013) and in accordance with the Medical Research Involving Human Subjects Act (WMO). TMS procedures will be performed according to expert guidelines on safety and recommendations for TMS use in healthy subjects and patient populations.[2]

11.2 Recruitment and consent

Patients potentially eligible for participation will be approached by the treating rehabilitation physician. The treating rehabilitation will ask the patient whether the local investigator can approach him or her. Patients will be screened for eligibility by the local investigator based on the inclusion and exclusion criteria, which includes eligibility according to the TMS safety questionnaire. If the patient is eligible, the local investigator explains the study procedures and provides the eligible patient with the patient information letter. The local investigator encourages the patient to read the information, discuss the content with friends or relatives and to write down questions that may arise. The patient will be given time to consider participation. During a second consultation with the local investigator, the investigator will give the patient the opportunity to ask questions, after which the patient can provide written informed consent. In case the patient does not have the capacity to provide written informed consent (e.g., due to upper limb impairments or aphasia), his/her representative may provide written informed consent. As soon as the patient has regained capacity, he/she will be asked for written informed consent. The patient will be provided with a copy of the signed informed consent.

11.3 Objection by minors or incapacitated subjects (if applicable)

Not applicable.

11.4 Benefits and risks assessment, group relatedness

Data from our phase II trial (B-STARS) show that cTBS treatment leads to a clinically relevant improvement in upper limb recovery, with 17% (n 60; 9.6 points; 95%CI 1.2–17.9) additional recovery of upper limb activity, as measured with the ARAT three months after stroke.[3] This additional recovery led to a significant improvement in activity and participation (of similar magnitude) and a reduction in the length of stay at the rehabilitation center by 18 days on average.[3]

cTBS treatment only led to mild side effects, such as headache (<4%) and muscle pain (<1%) and was reported to be tolerable and comfortable.[3] The risk of inducing an epileptic seizure is very small (0.02%) and consequences of an epileptic seizure can be mitigated by adequate training of personnel.[10] The risk of experiencing an epileptic seizure in the early phase after stroke (not induced by TMS) varies from 2 to 12%.[11] Therefore, rehabilitation centers have protocols in place. TMS-therapists are instructed to adhere to local protocols in case of an epileptic seizure. We are aware that including patients in a clinical trial in the early stages of recovery imposes a burden on them. However, evidence shows that treatment is most likely to be effective in the early post-stroke phase.[4]

Therefore, cTBS treatment is likely to have a clinically relevant benefit, while the risk and burden of participation are negligible.

11.5 Compensation for injury

The sponsor/investigator has liability insurance in accordance with article 7 of the WMO.

The sponsor (also) has an insurance which is in accordance with the legal requirements in the Netherlands (Article 7 WMO). This insurance provides cover for damage to research subjects through injury or death caused by the study.
The insurance applies to the damage that becomes apparent during the study or within 4 years after the end of the study.

11.6 Incentives (if applicable)

Patients do not have any special incentive to participate in this study.

Travel costs for study participation are compensated for when patients are discharged from the rehabilitation center during follow-up.

12. ADMINISTRATIVE ASPECTS, MONITORING AND PUBLICATION

12.1 Handling and storage of data and documents

All personal data will be handled confidentially according to the EU General Data protection regulation (GDPR) and the Dutch General data Protection Regulation (AVG). Each participant will be allocated an identification code as part of the randomization procedure. Only the local investigator has access to the key table that links the identification code to the participant’s personal information. The key table will be encrypted and stored on a local hard drive in the rehabilitation center.

Non-personal study data will be entered into CASTOR through an eCRF by the investigator who included the participant, or by his/her local representative. The participant's email address will be submitted in CASTOR separately. The email address is used to send out medical consumption and productivity questionnaires 6 months after stroke.

Informed consents, questionnaires, and forms on treatment parameters and motor function tests and the EMG data will be stored at the local archive of the rehabilitation center and will be safeguarded by the local investigator. Data from the paper forms will be entered into CASTOR through an eCRF by the investigator who performed the treatment or assessment, or by a representative. Anonymous EMG data will be transferred to the UMC Utrecht through SURFfilesender using encryption. The raw data will be stored for as long as the data is used for research purposes and for 15 years minimally.

The combined dataset in CASTOR from all participating centers can only be accessed by the coordinating investigators. Local investigators will be provided with a subset of the data in CASTOR that was acquired at their study site.

12.2 Monitoring and Quality Assurance

This study has a negligible risk, based on the risk classification of the Dutch Federation of University Medical Centers (NFU). Intensity of monitoring is based on the risk classification. Monitoring will be done by an independent and qualified monitor. This person is not involved in the design and execution of the study. Details can be found in a separate monitoring plan (K6).

12.3 Amendments

Amendments are changes made to the research after a favorable opinion by the accredited METC has been given. All amendments will be notified to the METC that gave a favorable opinion.

12.4 Annual progress report

The sponsor/investigator will submit a summary of the progress of the trial to the accredited METC once a year. Information will be provided on the date of inclusion of the first subject, numbers of subjects included and numbers of subjects that have completed the trial, serious adverse events/ serious adverse reactions, other problems, and amendments.

12.5 Temporary halt and (prematurely) end of study report

The investigator/sponsor will notify the accredited METC of the end of the study within a period of 8 weeks thereafter. The end of the study is defined as the last patient's last visit. The sponsor will notify the METC immediately of a temporary halt of the study, including the reason of such an action. In case the study is ended prematurely, the sponsor will notify the accredited METC within 15 days, including the reasons for the premature termination. Within one year after the end of the study, the investigator/sponsor will submit a final study report with the results of the study, including any publications/abstracts of the study, to the accredited METC.

12.6 Public disclosure and publication policy

The trial is registered in a public clinical trial registry (clinicaltrials.gov) with identifier: NCT06265766. Results of this trial will be disclosed and published as an open access article in peer-reviewed international scientific journals.

13. STRUCTURED RISK ANALYSIS

13.1 Potential issues of concern

a. Level of knowledge about mechanism of action

A single TMS pulse can activate cortical neurons through electromagnetic induction. Repetitive delivery of TMS pulses can increase or reduce activity and excitability in the stimulated area depending on the stimulation paradigm, through long-term potentiation or depression (LTP/LTD) mechanisms, respectively.[12] Stimulation paradigms such as low-frequency stimulation and continuous theta burst stimulation have been shown to reduce activity and excitability in the stimulated area. The effects of stimulation are not restricted to the stimulated area, but spread to connected brain regions.[13]

The exact mechanism of rTMS treatment in promoting upper limb recovery is unclear. Previous neuroimaging and neurophysiological studies indicate that a motor stroke affects the balance in bilateral M1 activity, with the magnitude of the imbalance correlating with the level of impairment.[14] Based on this evidence, it has been hypothesized that reducing contralesional M1 activity with inhibitory rTMS can promote recovery of the affected upper limb. Our phase II trial data show that inhibitory rTMS of the contralesional M1 promotes recovery of the upper limb.[3] We also showed that treatment was associated with an increase in ipsilesional M1 excitability directly after 10 treatment sessions, providing a potential treatment mechanism.

b. Previous exposure of human beings with the test product(s) and/or products with a similar biological mechanism

TMS was invented in 1985 by Anthony Barker, after which it has been used to safely stimulate and study human motor cortex for over 35 years. TMS has been used repetitively for therapeutic purposes since its approval for the treatment of depression in 2008. Since then, several other clinical applications have been introduced, resulting in an abundance of TMS safety data.

c. Can the primary or secondary mechanism be induced in animals and/or in *ex-vivo* human cell material?

Yes, promotion of upper limb recovery with contralesional inhibitory rTMS can be studied in animals but not in ex-vivo human cell material.

d. Selectivity of the mechanism to target tissue in animals and/or human beings

TMS using a figure-of-eight coil results in a relatively focal effective stimulation area of roughly 1cm^3 of cortical tissue. This allows specific targeting of the M1 hand area, but does not rule out activation of neurons in premotor or sensory areas.

e. Analysis of potential effect

Data from our phase II trial (B-STARS) indicate that cTBS treatment results in 17% (n = 60; 9.6 points; 95%CI 1.2–17.9) additional recovery of upper limb function, as measured with the ARAT three months after stroke.[3] This improvement exceeds the minimal clinically important difference of 10%. This additional recovery led to a significant improvement in activity and participation (of similar magnitude) and a reduction in the length of stay at the rehabilitation center by 18 days on average. In the current phase III trial, the potential effect will be analyzed with a similar selection of measurement tools to assess upper limb impairment and activity, disability and dependence and quality of life.

Our phase II trial data show that inhibitory rTMS only led to mild side effects, such as headache (<4%) and muscle pain (<1%).[3] The current trial will assess side-effects of cTBS during treatment and within 1 week thereafter.

f. Pharmacokinetic considerations

Not applicable.

g. Study population

The study population consists of stroke patients who are in the early subacute phase of their recovery (within 3 weeks after stroke onset). This means that the study population consists of patients who are in the early stages of their recovery from a severe health condition. However, the early start of treatment is required for optimal treatment efficacy. A meta-analysis from our group and a guidelines paper show that treatment efficacy is likely to be optimal when rTMS treatment is started within 1 month after stroke onset.[4] Our phase II trial data, in which treatment was started within 3 weeks after stroke onset confirm these findings.[3] Moreover, the rTMS treatment induces only minimal strain on the subjects, due to the short duration (10 minutes preparation and 40s treatment duration) and comfortable setting (patients are seated in a reclining chair and don’t have to perform an activity). Interviews with a selected number of subjects of the phase II trial revealed that cTBS was very tolerable and in some cases even provided a moment of relaxation in an otherwise busy rehabilitation program.[15]

h. Interaction with other products

rTMS treatment must be avoided in patients with implants which are close to the scalp, such as cochlear implants or neurostimulators.[16] The TMS safety questionnaire is designed to identify such cases.[16]

rTMS treatment poses an increased risk of inducing an epileptic seizure in case of an interaction with products with a significant seizure-threshold-lowering potential. These have been well-described in TMS guidelines.[16]

i. Predictability of effect

In the phase II trial, we included a broad range of patients with a first-ever ischemic stroke or intracerebral hemorrhage that resulted in unilateral upper extremity paresis. [3] A meta-analysis shows that treatment efficacy is higher when treatment is started within the first month post-stroke with a mean difference of 9.3 points on the Fugl-Meyer Upper Extremity, compared to a mean difference of 1.14 points between 1 and 3 months and after 3 months post-stroke.[4]

j. Can effects be managed?

cTBS treatment has a risk of 0.02% per cTBS session of inducing an epileptic seizure.[17] This was not observed in our phase II trial.[3] The consequences of inducing an epileptic seizure can be mitigated by adequate training of personnel. Other side effects of cTBS treatment are minor (headache, muscle pain) and wear off within a few hours.[10]

13.2 Synthesis

Data from our phase II trial (B-STARS) shows that cTBS treatment leads to a clinically relevant improvement in upper limb recovery, with 17% (n = 60; 9.6 points; 95%CI 1.2–17.9) additional recovery of upper limb activity, as measured with the ARAT three months after stroke.[3] This additional recovery led to a significant improvement in activity and participation (of similar magnitude) and a reduction in the length of stay at the rehabilitation center by 18 days on average.[3]

cTBS treatment only led to mild side effects, such as headache (<4%) and muscle pain (<1%) and was reported to be tolerable and comfortable.[3] The risk of inducing an epileptic seizure is rare (0.02%) and consequences of an epileptic seizure can be mitigated by adequate training of personnel.[17]

In conclusion, the potential benefits of cTBS treatment are substantial and the potential risks are minor and manageable.

14. REFERENCES

1. Lieshout ECC van, van de Port IG, Dijkhuizen RM, Visser-Meily JMA. Does upper limb strength play a prominent role in health-related quality of life in stroke patients discharged from inpatient rehabilitation? *Top Stroke Rehabil*. 2020;27(7):525-533.
2. Lefaucheur JP, Aleman A, Baeken C, et al. Evidence-based guidelines on the therapeutic use of repetitive transcranial magnetic stimulation (rTMS): An update (2014–2018). *Clinical Neurophysiology*. 2020;131(2):474-528. doi:10.1016/j.clinph.2019.11.002
3. Vink JJT, van Lieshout ECC, Otte WM, et al. Continuous theta-burst stimulation of the contralesional primary motor cortex for promotion of upper limb recovery after stroke: a randomized controlled trial. *Stroke*. Published online 2023.
4. van Lieshout ECC, van der Worp HB, Visser-Meily JMA, Dijkhuizen RM. Timing of Repetitive Transcranial Magnetic Stimulation Onset for Upper Limb Function After Stroke: A Systematic Review and Meta-Analysis. *Front Neurol*. 2019;10(1269):1-16. doi:10.3389/fneur.2019.01269
5. Hofmeijer J, Ham F, Kwakkel G. Evidence of rTMS for Motor or Cognitive Stroke Recovery: Hype or Hope? *Stroke*. 2023;54(10):2500-2511.
6. Faul F, Erdfelder E, Lang AG, Buchner A. G* Power 3: A flexible statistical power analysis program for the social, behavioral, and biomedical sciences. *Behav Res Methods*. 2007;39(2):175-191.
7. Page SJ, Fulk GD, Boyne P. Clinically important differences for the upper-extremity Fugl-Meyer Scale in people with minimal to moderate impairment due to chronic stroke. *Phys Ther*. 2012;92(6):791-798.
8. Gladstone DJ, Danells CJ, Black SE. The fugl-meyer assessment of motor recovery after stroke: A critical review of its measurement properties. *Neurorehabil Neural Repair*. 2002;16(3):232-240. doi:10.1177/154596802401105171
9. Rodríguez A, Amaya-Pascasio L, Gutiérrez-Fernández M, García-Pinteño J, Moreno M, Martínez-Sánchez P. Non-invasive brain stimulation for functional recovery in animal models of stroke: A systematic review. *Neurosci Biobehav Rev*. 2024;156. doi:10.1016/j.neubiorev.2023.105485
10. Oberman L, Edwards D, … MEJ of C, 2011 undefined. Safety of theta burst transcranial magnetic stimulation: a systematic review of the literature. *ncbi.nlm.nih.govL Oberman, D Edwards, M Eldaief, A Pascual-LeoneJournal of Clinical Neurophysiology, 2011•ncbi.nlm.nih.gov*. Accessed January 2, 2024. https://www.ncbi.nlm.nih.gov/pmc/articles/PMC3260517/
11. Holtkamp M, Beghi E, Benninger F, et al. European Stroke Organisation guidelines for the management of post-stroke seizures and epilepsy. *Eur Stroke J*. 2017;2(2):103-115.
12. Cirillo G, Di Pino G, Capone F, et al. Neurobiological after-effects of non-invasive brain stimulation. *Brain Stimul*. 2017;10(1):1-18. doi:10.1016/j.brs.2016.11.009
13. Vink JJJTT, Mandija S, Petrov PIPI, van den Berg CATCAT, Sommer IECIEC, Neggers SFWSFW. A novel concurrent TMS-fMRI method to reveal propagation patterns of prefrontal magnetic brain stimulation. *Hum Brain Mapp*. 2018;39(11). doi:10.1002/hbm.24307
14. Boddington LJ, Reynolds JNJ. Targeting interhemispheric inhibition with neuromodulation to enhance stroke rehabilitation. *Brain Stimul*. 2017;10(2):214-222. doi:10.1016/j.brs.2017.01.006
15. Van Lieshout ECC, Jacobs LD, Pelsma M, Dijkhuizen RM, Visser-Meily JMA. Exploring the experiences of stroke patients treated with transcranial magnetic stimulation for upper limb recovery: A qualitative study. *BMC Neurol*. 2020;20(1). doi:10.1186/s12883-020-01936-5

16. Rossi S, Antal A, Bestmann S, et al. Safety and recommendations for TMS use in healthy subjects and patient populations, with updates on training, ethical and regulatory issues: Expert Guidelines. *Clinical Neurophysiology*. 2021;132(1):269-306. doi:10.1016/j.clinph.2020.10.003
17. Oberman L, Edwards D, Eldaief M, Pascual-Leone A. Safety of theta burst transcranial magnetic stimulation: a systematic review of the literature. *Journal of Clinical Neurophysiology*. 2011;28(1):67.
18. Balami JS, Chen RL, Grunwald IQ, Buchan AM. Neurological complications of acute ischaemic stroke. *Lancet Neurol*. 2011;10(4):357-371.
19. Balami JS, Buchan AM. Complications of intracerebral haemorrhage. *Lancet Neurol*. 2012;11(1):101-118.
20. Hesse K, Fulton RL, Abdul-Rahim AH, et al. Characteristic adverse events and their incidence among patients participating in acute ischemic stroke trials. *Stroke*. 2014;45(9):2677-2682.
21. Kumar S, Selim MH, Caplan LR. Medical complications after stroke. *Lancet Neurol*. 2010;9(1):105-118.

Appendix A

Rehabilitation center	Local investigator
De Hoogstraat	**Mirjam Kouwenhoven**<br>m.kouwenhoven@dehoogstraat.nl
Basalt Den Haag	**Lotte Mulder**<br>l.mulder@basaltrevalidatie.nl
Adelante	**Carlijn Wiertz**<br>carlijn.wiertz@adelantegroep.nl
MRC Aardenburg	**Lisa Kruisheer**<br>EM.Kruisheer@mrcdoorn.nl
Heliomare	**Deborah Wit**<br>d.wit@heliomare.nl
Vogellanden	**Bente Visser**<br>b.visser@vogellanden.nl
Reade	**Celine Timmermans**<br>c.timmermans@reade.nl
Merem	**Marijke van Bloemendaal en Karin Peek**<br>bstars@merem.nl
Tolbrug	**Cynthia Klinkers**<br>C.Klinkers@tolbrug.nl
Revalidatie Friesland	**Wietske Rienstra**<br>w.rienstra@revalidatie-friesland.nl
Sint Maartenskliek	**Nelleke Kooiman**<br>n.kooiman@maartenskliniek.nl
Revant	**Marissa Riemens**<br>Marissa.Riemens@revant.nl
UMCG	**Henk Meulenbelt**<br>h.e.j.meulenbelt@umcg.nl
Basalt Leiden	**Annelies Mantje**<br>a.mantje@basaltrevalidatie.nl
Libra Leijpark	**Iris Habets**<br>i.habets@libranet.nl
Libra Blixembosch	**Daphne Stranders**<br>d.stranders@libranet.nl

Appendix B – common (potentially) serious adverse events after stroke

Based on:[18–21]

Cardiac<br>Angina<br>Arrhythmia<br>Atrial fibrillation<br>Angina pectoris<br>Bradycardia<br>Cardiac arrest<br>Cardiomyopathy<br>Heart failure<br>Myocardial infarction<br>Tachycardia	Pulmonary<br>Aspiration<br>Bronchitis<br>Chronic obstructive pulmonary disease<br>Dyspnea<br>Obstructive sleep apnea<br>Oxygen desaturation<br>Pneumonia<br>Pulmonary embolism<br>Respiratory failure / arrest<br>Respiratory tract infection	Gastro-intestinal<br>Constipation<br>Dysphagia<br>Fecal incontinence<br>Gastro-intestinal hemorrhage<br>Ileus<br>Melena<br>Mucosal irritation<br>Nausea<br>Rectal hemorrhage<br>Stress ulcer<br>Vomiting
Central nervous system<br>Delirium<br>Depression<br>Epileptic seizure<br>Headache<br>Hydrocephalus<br>Recurrent ischemic stroke<br>Recurrent intracerebral hemorrhage<br>Recurrent stroke<br>Retinal ischemia<br>Sleep disorder<br>Status epilepticus<br>Spasticity<br>Transient ischemic attack<br>Transient monocular blindness	General / other<br>Anemia<br>Arterial hypertension<br>Arterial hypotension<br>Deep vein thrombosis<br>Dehydration<br>Fall (and consequences)<br>Fatigue<br>Fever<br>Hematuria<br>Hip fracture<br>Hyperglycemia<br>Infections<br>Pain<br>Pressure sore<br>Renal failure<br>Sepsis<br>Syncope<br>Undernutrition<br>Urinary incontinence<br>Urinary tract infection	

Appendix C – Trial organisation

The Executive committee of the trial consists of the trial's central PIs and coordinating investigators. They meet regularly, discuss trial progress and prepare information for the Steering committee.

Statistical Analysis Plan

B-STARS 2: Brain Stimulation for Arm Recovery after Stroke 2. A phase III, randomized, double-blind, sham-controlled, clinical trial.

Dutch trial register	NL85511.041.24
Clinicaltrials.gov Identifier	NCT06265766
Funder	Dutch National Health Care Institute
Sponsor	University Medical Center Utrecht
Version	3.0
Date	16-05-2025
Principal investigator	Prof. Dr. J.M.A. (Anne) Visser-Meily<br>j.m.a.visser-meily@umcutrecht.nl
Project leaders	Prof. Dr. R.M. (Rick) Dijkhuizen<br>r.m.dijkhuizen@umcutrecht.nl<br>Prof. Dr. H.B. (Bart) van der Worp<br>h.b.vanderworp@umcutrecht.nl
Coordinating investigators	Dr. J.J.T. (Jord) Vink<br>j.j.vink-5@umcutrecht.nl<br>T.A. (Tessa) Verhoeff<br>t.a.verhoeff-2@umcutrecht.nl
Trial statistician/epidemiologist	Dr. W.M. (Wim) Otte<br>w.m.otte@umcutrecht.nl
Independent statistician	Dr. R.P.A. (Ruben) van Eijk<br>r.p.a.vaneijk-2@umcutrecht.nl

Table of contents

1. Study objectives 3
2. Study design 3
3. Primary outcome 4
4. Secondary outcomes 5
5. Overall data and statistical analysis principals 6
5.1. Blinding & data lock 6
5.2. Handling of missing data 7
5.3. Quality control 7
6. Primary estimand 7
6.1. Patient population 7
6.2. Primary endpoint 7
6.3. Population level summary 8
6.4. Treatment condition 8
6.5. Intercurrent event handling 8
7. Analyses to be performed 8
7.1. Retention 8
7.2. Baseline data 8
7.3. Adherence to allocated treatment 9
7.4. Primary analysis 9
7.5. Secondary analyses 9
7.6. Sensitivity analyses 10
7.7. Subgroup analyses 10
7.8. Safety 10
8. Change log 11
9. References 11

1. Study objectives

Primary Objective:

- To assess whether 10 sessions of cTBS of the contralesional primary motor cortex (M1) combined with regular care upper limb training, started within three weeks after stroke onset and continued every working day for two weeks, reduce upper limb impairment at 90 days after stroke, compared to sham stimulation.

Secondary Objective(s):

To assess whether 10 sessions of cTBS of the contralesional M1 combined with regular care upper limb training, started within three weeks after stroke onset and continued every working day for two weeks:

- improve upper limb activity, manual dexterity and quality of life, and reduces disability and dependence at 90 days and 12 months after stroke, compared to sham stimulation;
- reduce upper limb impairment at 12 months after stroke, compared to sham stimulation;
- are cost-effective compared to sham stimulation;
- reduce excitability of the contralesional M1 during the 2-week treatment period, compared to sham stimulation;
- increase ipsilesional M1 excitability after 10 treatment sessions, compared to sham stimulation.

2. Study design

B-STARS2 is a multi-centre, double-blind, randomised, sham-controlled, clinical trial. Participants will be recruited from 16 rehabilitation centres in the Netherlands over a period of 51 months (Q2 of 2024 to Q4 of 2028). The total duration of follow-up for each participant is until one year after stroke. The total study duration is 63 months.

Baseline assessment will be performed before the start of the intervention, which must occur within 21 days after stroke onset. Follow-up assessments will be performed at 90 days (+/- 7 days) and 12 months (+/- 14 days) post-stroke (Figure 1).

The intervention consists of ten daily sessions of active or sham cTBS, delivered over the contralesional primary motor cortex, started within 21 days post-stroke and combined with regular care upper limb therapy.

Participants are randomly assigned in a 1:1 ratio to one of the two treatment groups (group A or B) using a web-based data management service. Treatment allocation is stratified by rehabilitation centre and the ability to extend one or more fingers of the affected hand.

An independent DSMB is monitoring the safety of participants of the trial. The DSMB meets at least annually. An independent statistician will perform unblinded interim analyses on all reported (serious) adverse events after 150 and 300 patients have completed the 90 days post-stroke follow-up. The independent statistician will report the outcome of the interim analyses directly to the DSMB, as well as listings of all SAE reports and unblinded aggregate summaries of data by treatment group for review in closed meetings. Feedback, blind to treatment, will be provided in written conclusions to the B-STARS2 project leaders.

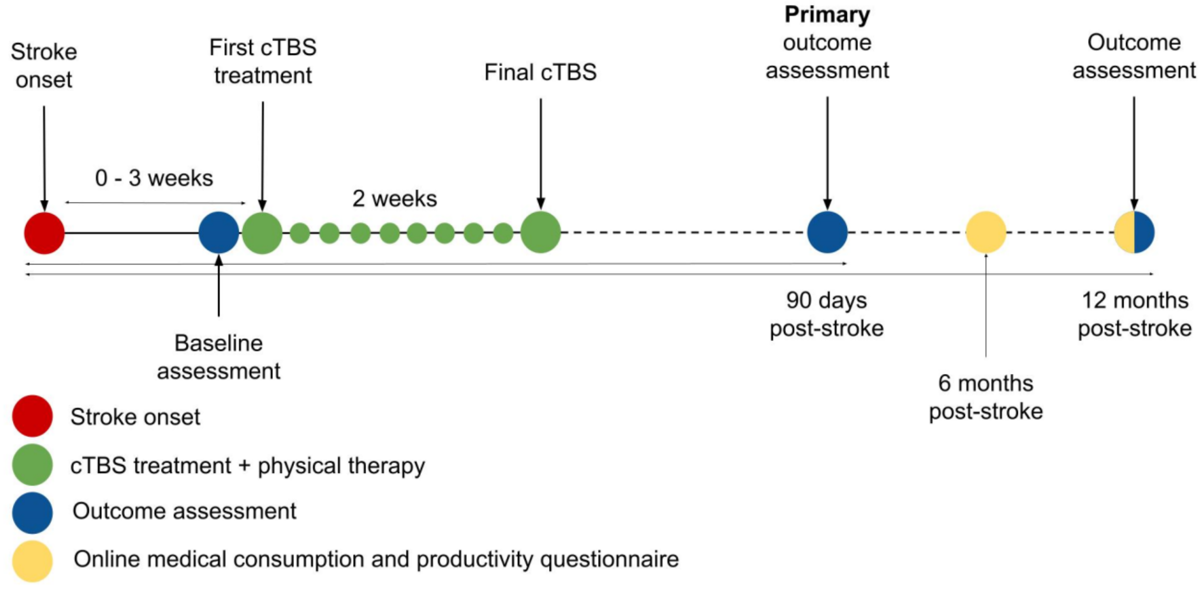

Figure 1. Study timeline.

3. Primary outcome

The primary outcome is the score on the Fugl-Meyer Upper Extremity Assessment (FM-UE) at 90 days post-stroke (Table 1). The FM-UE is a reliable and valid motor performance test consisting of 33 tasks that assesses upper limb motor impairment on a scale from 0 to 66 points, with a higher score indicating less impairment. The FM-UE will be assessed by certified assessors. Before

certification, assessors will receive on-site training by the team of coordinating investigators and must successfully complete an FM-UE exam.

4. Secondary outcomes

Secondary outcomes are the score on the FM-UE at 12 months post-stroke and scores on the Action Research Arm Test, Nine Hole Peg Test, modified Rankin Scale, Barthel Index, hand and participation sections of the Stroke Impact Scale, the EuroQol-5D-5L at 90 days and 12 months post-stroke. Additional secondary outcomes are length of stay in the rehabilitation centre, change in ipsilesional and contralesional excitability and cost effectiveness. Secondary outcomes are described in Table 1.

Table 1. Outcome assessments

Assessment	Timepoint	Description	Abbreviation
**Primary outcome**			
Fugl-Meyer – Upper Extremity	90 days	Upper extremity impairment test	FM-UE
**Secondary outcomes**			
Fugl-Meyer – Upper Extremity	12 months	Upper extremity impairment test	FM-UE
Action Research Arm Test	90 days, 12 months	Upper extremity activity test	ARAT
Nine Hole Peg Test	90 days, 12 months	Manual dexterity test	NHPT
Modified Rankin Scale	90 days, 12 months	Disability and dependence questionnaire	mRS
Barthel Index	90 days, 12 months	Activities of daily living questionnaire	BI
Stroke Impact Scale – Hand	90 days, 12 months	Hand function questionnaire	SIS-hand
Stroke Impact Scale - participation	90 days, 12 months	Participation questionnaire	SIS-par

EuroQol - 5 dimensions	90 days, 12 months	Quality of life questionnaire	EQ-5D-5L
Length of stay		Length of stay in the rehabilitation center	LOS
Contralesional excitability	Before the 1st, 6th and 10th cTBS session	Resting motor threshold of the contralesional M1	cRMT
Ipsilesional excitability	Before the 1st and 10th cTBS session.	Resting motor threshold of the ipsilesional M1	iRMT
Contralesional inhibition	Before and after the 1st, 6th and 10th cTBS session	Ratio between the average motor-evoked potential amplitude before and after cTBS.	CI

5. Overall data and statistical analysis principals

The analysis and reporting of the trial will be in accordance with CONSORT guidelines. This statistical analysis plan (SAP) specifies: (i) Hypotheses to be tested; (ii) Treatment effects to be estimated in order to satisfy the primary and secondary objectives of this trial; (iii) Technical description of the statistical methodology and procedures for performing the statistical analysis of outcome measures and SAE data; (iv) Primary, secondary, and sensitivity analyses; and (v) Subgroup analyses. The SAP will be signed off by the trial Executive Committee and the trial statistician and then published. The final statistical analyses will be performed once recruitment has ceased, final follow-up has been completed, final data have been checked and any errors corrected, and the database has been locked.

5.1. Blinding & data lock

Sham cTBS will be performed using a sham TMS coil, which looks identical to an active TMS coil but does not produce effective stimulation. The sham and active TMS coils will be labelled with a code (A/B) by the coordinating investigator who is not involved in any of the TMS treatments. The TMS therapist performs randomization and patients will be allocated to receive treatment with coil A or B, depending on their group allocation (A/B). Therefore, the TMS therapists and patients are

unaware of treatment allocation. Blinding of the patient and therapist will be maintained until the last patient has completed the final follow-up (at 12 months post-stroke).

Only one of the coordinating investigators can link treatment allocation to the active or sham condition. The independent trial statistician will combine data on treatment allocation with the clinical data to report to the data safety monitoring board (DSMB). The treatment allocation code will not be broken until the database has been locked, which is after the last patient has completed the final follow-up and all data have been cleaned. Cleaning of the data will be performed by a coordinating investigator who is unaware of treatment allocation.

5.2. Handling of missing data

Missing data due to loss to follow-up are assumed to be missing at random. Missing data will be reported. Prior to analysis, missing data is imputed using multiple imputation.[1] Imputation will be performed using multiple imputation generating 500 datasets to account for inherent uncertainty, performed separately for each treatment group. This will be described in the results section of the manuscript.

5.3. Quality control

Before database lock and unblinding, summary tables will be verified, potential errors will be corrected if needed and full analyses will be performed based on dummy randomization codes. Statistical analyses, including R code, log and output will be peer reviewed to ensure accuracy of the analyses.

6. Primary estimand

Secondary endpoints will use a similar framework for the main estimand.

6.1. Patient population

The patient population is based on the intention-to-treat principle and consists of all subjects who are randomized to active or sham cTBS and started at least one cTBS session (ITT1), analysed according to the treatment group they were randomized to.

6.2. Primary endpoint

The primary endpoint is the change from baseline in FM-UE score at 90 days post-stroke.

6.3. Population level summary

The difference in the mean FM-UE change from baseline to 90 days post-stroke between active and sham cTBS.

6.4. Treatment condition

Active cTBS compared to sham cTBS.

6.5. Intercurrent event handling

Death: Missing data in case of death will be imputed with an FM-UE score of 0.

Treatment Discontinuation: Data collected after treatment discontinuation will be included and analysed according to the treatment group to which the participant was randomized, except if treatment was discontinued before the first cTBS session.

7. Analyses to be performed

7.1. Retention

We will provide a CONSORT flow diagram, reporting the number of patients who were randomized, treated, adhered to and completed follow-up by treatment group and report reasons for withdrawal.

7.2. Baseline data

We will summarize baseline characteristics per treatment group without formal statistical testing. The following will be presented: Age, sex, ethnicity, living arrangement, employment status, status at baseline (FM-UE, ARAT, NHPT, BI, mRS, SIS, EQ-5D-5L), NIHSS at hospital admission, time between stroke onset and start of cTBS, lesion type (ischaemic stroke or intracerebral haemorrhage, lesion location (cortical subcortical or brainstem), right hemisphere affected and dominant hemisphere affected), electrophysiological status (MEP presence), stratification factor (ability to extend one or more fingers), acute intervention, time between stroke onset and start of upper limb therapy and time between stroke onset and admission to rehabilitation centre, cardiovascular risk factors (diabetes mellitus, hypertension and smoking).

7.3. Adherence to allocated treatment

We will summarize the number of completed cTBS sessions per treatment group, descriptively.

7.4. Primary analysis

The primary outcome is the FM-UE score at 90 days poststroke. The FM-UE reflects upper limb impairment on an ordinal scale from 0 (maximum impairment) to 66 points (no impairment).

The primary analysis is performed using a linear mixed model. Analysis will be performed in the primary estimand, including all randomized subjects who started at least 1 cTBS session. The outcome variable is the FM-UE score at 90 days poststroke. The model includes fixed effects for baseline FM-UE score; stratification factor (ability versus no ability to extend one or more fingers), age, gender, stroke subtype (ischemic stroke vs. intracerebral haemorrhage) and treatment (sham cTBS; active cTBS) and random effects for rehabilitation centre.

7.5. Secondary analyses

The FM-UE, ARAT, NHPT, mRS, SIS-hand and SIS-participation and EQ-5D-5L at 90 days and 12 months post-stroke will be assessed using a mixed model for repeated measures or cumulative link mixed model, depending on the type of outcome measure. The outcome variable is the secondary outcome. The model includes fixed effects for the corresponding baseline scores; the stratification factor (ability versus no ability to extend one or more fingers), age, gender, stroke subtype (ischemic stroke vs. Intracerebral haemorrhage), visit (90 days; 12 months) and the interaction of treatment (sham cTBS; active cTBS) by visit and random effects for rehabilitation centre.

The length of stay will be analysed using a two-sample independent t-test.

Ipsilesional excitability will be analysed using a linear mixed model. The outcome variable is the ipsilesional RMT after the final cTBS session. The model includes fixed effects for the ipsilesional RMT before the first cTBS session, stratification factor (ability versus no ability to extend 1 or more fingers) and treatment (sham cTBS; active cTBS) and random effects for rehabilitation centre.

Contralesional excitability before the 1st, 6th and 10th cTBS session will be analysed using a mixed model for repeated measures. The outcome variable is the contralesional RMT determined before the 1st, 6th and 10th cTBS session. The model includes fixed effects for baseline RMT, cTBS session

number (1, 6 or 10) and type of treatment (sham cTBS; active cTBS) and random effects for rehabilitation centre.

Contralesional excitability after the 1st, 6th and 10th cTBS session will be analysed using a mixed model for repeated measures. The outcome variable is the contralesional MEP amplitude after cTBS. The model includes fixed effects for the contralesional MEP amplitude before cTBS, cTBS session (1, 6 or 10) and treatment (sham cTBS; active cTBS) and random effects for rehabilitation centre.

7.6. Sensitivity analyses

A first sensitivity analysis will be performed in the per-protocol population, defined as cases in which a valid FM-UE score could assessed at 90 days post-stroke, in which at least eight cTBS sessions were performed, that were unaffected by a recurrent stroke and in which no major protocol violation was identified. A second sensitivity analysis will be performed on complete cases, without imputation of missing data. A third sensitivity analysis will be performed based on the intention-to-treat population, consisting of all randomized subjects (ITT2). In case of unbalanced baseline characteristics, additional sensitivity analyses may be performed that include the unbalanced baseline characteristic as a fixed effect in the generalized linear mixed model described in section 7.4.

7.7. Subgroup analyses

Predefined subgroup analyses will be based on baseline FM-UE extremity score (FM ≤ 20 versus FM > 20); stroke subtype (ischemic stroke versus intracerebral haemorrhage); stroke location (cortical versus subcortical versus brainstem) and time to start of treatment (within 14 days versus 15 to 21 days). These analyses will be performed by including an interaction term between treatment and the previously described parameters in the linear regression model described in section 7.4.

7.8. Safety

We will report (serious) adverse events tabulated per treatment group.

8. Change log

Version	Changes	Date
2	- Section added on blinding and data lock<br>- Additional sensitivity analyses (complete case and ITT2) added	14-10-2024
3	Based on reviewer recommendations:<br>- Gender added as fixed effect in primary and secondary analyses<br>- Per protocol population definition adjusted to: ‘cases in which a valid FM-UE score could assessed at 90 days post-stroke, in which at least eight cTBS sessions were performed, that were unaffected by a recurrent stroke and in which no major protocol violation was identified.’	16-05-2024

9. References

1. Buuren V. Flexible Imputation of Missing Data Second edition. 2018;29.

Cost Utility Analysis and Budget Impact Analysis Plan

B-STARS 2: Brain Stimulation for Arm Recovery after Stroke 2. A phase III, randomized, double-blind, sham-controlled, clinical trial.

Dutch trial register	NL85511.041.24
Clinicaltrials.gov Identifier	NCT06265766
Funder	Dutch National Health Care Institute
Sponsor	University Medical Centre Utrecht
Version	2.0
Date	14-10-2024
Coordinating investigators	Dr. J.J. (Jord) Vink<br>j.j.vink-5@umcutrecht.nl<br>T.A. (Tessa) Verhoeff<br>t.a.verhoeff-2@umcutrecht.nl
Principal investigator	Prof. Dr. J.M.A. (Anne) Visser-Meily<br>j.m.a.visser-meily@umcutrecht.nl
Project leaders	Prof. Dr. R.M. (Rick) Dijkhuizen<br>r.m.dijkhuizen@umcutrecht.nl<br>Prof. Dr. H.B. (Bart) van der Worp<br>h.b.vanderworp@umcutrecht.nl
Trial health economist	Dr. Miriam van der Meulen<br>m.p.vandermeulen-16@umcutrecht.nl

Table of contents

Table of contents 2
1. Objectives 3
1.1. Efficacy objective 3
1.2. Health-economic objective 3
2. PICOTS 3
2.1. Patients 3
2.2. Intervention 3
2.3. Comparison 4
2.4. Outcomes 4
2.5. Time 4
2.6. Setting 5
3. Study design 5
4. Cost-utility analysis 6
4.1. Primary analysis 6
4.2. Scenario analyses 6
5. Budget Impact Analysis 7
6. Effects 7
7. Costs 8
7.1. Costs of the intervention 8
7.2. Clinical and outpatient rehabilitation costs 8
7.3. Other healthcare costs and informal care 8
7.4. Productivity costs 9
8. Questionnaires 9
9. Change log 9
10. References 9
Appendix A 10

1. Objectives

1.1. Efficacy objective

To assess whether 10 sessions of cTBS of the contralesional primary motor cortex (M1) combined with regular care upper limb training, started within three weeks after stroke onset and continued every working day for two weeks, reduces upper limb impairment at 90 days after stroke, compared to sham stimulation.

1.2. Health-economic objective

We will perform a cost-utility analysis from a societal perspective to assess whether 10 sessions of cTBS are cost-effective compared to sham cTBS in promoting upper limb recovery after stroke.

2. PICOTS

2.1. Patients

The patient population consists of patients with ischaemic stroke or intracerebral haemorrhage with unilateral upper limb impairment who are enrolled in a clinical rehabilitation programme in The Netherlands.

2.2. Intervention

The intervention consists of ten daily sessions of cTBS, delivered over the contralesional primary motor cortex, started within 21 days post-stroke. The intervention will be delivered by a physical or occupational therapist immediately before regular care upper limb therapy. A cTBS session consists of localization of the treatment target, determination of the treatment dose, and the actual stimulation paradigm. The intervention is intended to modulate neuronal activity in the motor cortex, to facilitate the effect of subsequent upper limb therapy.

2.3. Comparison

The cTBS intervention is an addition to regular care. The comparison consists of ten daily sessions of sham cTBS. Sham cTBS is delivered under the same conditions but using a sham TMS coil that does not produce effective stimulation. Sham cTBS is required to rule out a potential placebo effect induced by TMS.

2.4. Outcomes

Cost-effectiveness

The main outcome of this health economic evaluation is the integration of incremental costs and incremental quality adjusted life years (QALYs) of cTBS compared to usual care.

Costs

The costs of the intervention are subdivided into direct medical costs, costs for patients and families, and productivity costs. Medical costs include the costs of the intervention/procedure itself, costs of clinical and outpatient rehabilitation and other medical costs, such as hospitalizations or first-line physical therapy. These costs are described in section 6.1 to 6.3. Informal care and productivity costs are described in section 6.4.

Effects

The effects will be determined based on quality-adjusted life years (QALYs), combining quality of life with mortality data and are described in section 5.

2.5. Time

On average, stroke patients spend 8 to 12 weeks in clinical rehabilitation and recover around 70% of lost function within the first 3 months after stroke. Subsequently, patients spend an average of 3 months in outpatient rehabilitation. Thereafter, patients reintegrate into society and if applicable, reintegrate into the workforce. We will extrapolate the quality of life and costs at one-year post-stroke over longer periods of time to investigate the potential impact on long-term cost-effectiveness. At one-year post-stroke, most patients

have completed rehabilitation and recovery will have plateaued. Therefore, we will perform a scenario analysis with a time horizon of the cost-utility analysis of one year after stroke.

2.6. Setting

The intervention will be implemented in medical specialist rehabilitation centres. Health care professionals within these centres are not familiar with cTBS treatment, but are familiar with other medical devices, such as electrical stimulation. The iMTA questionnaires are adapted to the patient population to make sure that the reported data is applicable and relevant for the investigated setting.

3. Study design

B-STARS2 is a multi-centre, double-blind, randomized, sham-controlled, clinical trial. Participants will be recruited from 16 rehabilitation centres in the Netherlands over a period of 51 months (Q2 of 2024 to Q4 of 2028). The total duration of follow-up for each participant is until one year after stroke. The total study duration is 63 months.

The intervention consists of ten daily sessions of active or sham cTBS, delivered over the contralesional primary motor cortex, started within 21 days post-stroke and combined with regular care upper limb therapy.

The cost-utility analysis will be performed alongside the B-STARS2 trial. During the trial, data on patient-reported medical consumption and productivity losses will be acquired through digital questionnaires at 6- and 12-months post-stroke and quality of life will be assessed at 3- and 12-months post-stroke (Figure 1).

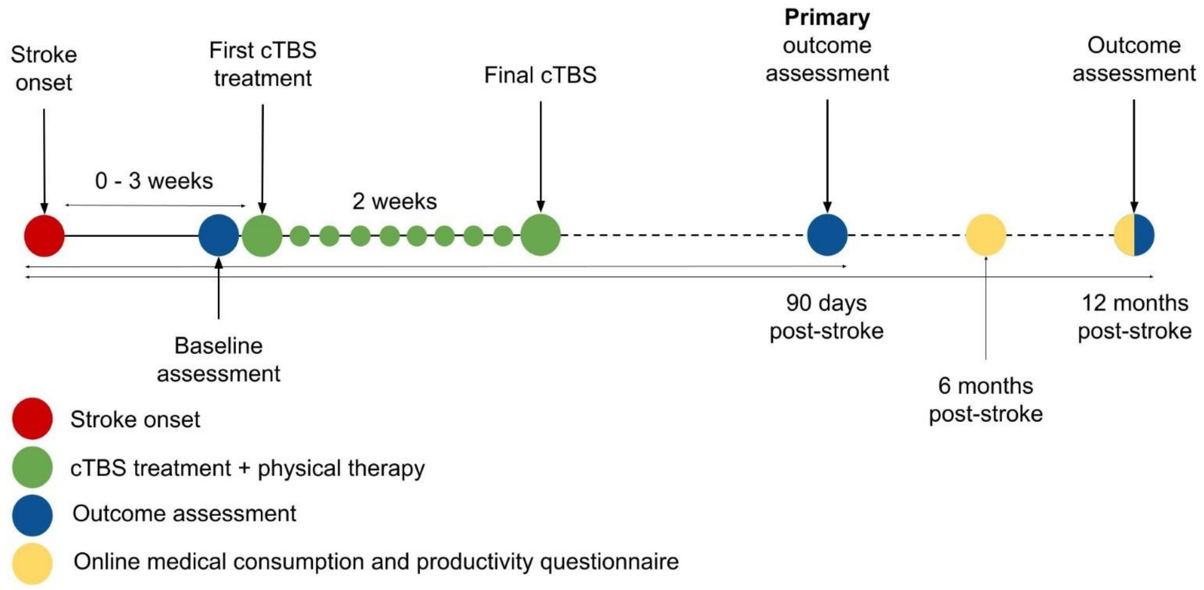

Figure 1. Study timeline.

4. Cost-utility analysis

4.1. Primary analysis

We will perform a cost-utility analysis from a societal perspective comparing active cTBS to sham cTBS. We will collect data on procedural costs, other direct medical costs, indirect costs, and patient-level quality of life. Missing data will be imputed with multiple imputation and the uncertainty of the economic evaluation will be assessed with bootstrapping techniques.

To be able to extrapolate trial findings and to perform other scenario analysis, a decision-analytical model (a decision tree) will be developed. In this decision tree, we will correlate EQ-5D-5L outcomes and costs to Fugl-Meyer Upper Extremity outcomes.

4.2. Scenario analyses

We will perform several scenario analyses. In the first scenario analysis, we will analyze the effects up to one-year post-stroke to investigate the impact on short-term cost-effectiveness. In a second scenario analysis we will investigate the potential impact of

sham cTBS on the cost-effectiveness analysis, as sham cTBS may have a placebo effect that complicates a comparison against the usual care situation. In a third scenario analysis, we will estimate cost-effectiveness if we exclude neuronavigation costs from the total costs of the intervention, because neuronavigation is only required for research purposes. A double-blind sham-controlled design requires blinding of the TMS therapist and the patient to the TMS coil used for treatment. Therefore, a separate coil is used to determine the treatment location and dose. Navigation is required to navigate the active or sham TMS coil to the target location without using stimuli. In a standard care situation, the same coil can be used for dose and location determination and treatment, eliminating the need for navigation.

5. Budget Impact Analysis

We will extrapolate the results from the cost utility analysis to all stroke patients who are eligible for the intervention. The budget impact analysis will be performed according to the Dutch BIA guideline and using the ZonMw calculation tool. The BIA will be performed in a probabilistic way and will take a 1) net budgetary framework healthcare (BKZ) perspective and a 2) health insurance / third party payer perspective.

6. Effects

Quality of life will be assessed with the EQ-5D-5L, within 3 weeks after stroke and at 3-, 6- and 12-months post-stroke. Quality of life is assessed at 3 and 6 months post-stroke, when most patients will have completed clinical rehabilitation. Quality of life will be assessed again at one-year post-stroke, when recovery has plateaued, and most patients will have ceased rehabilitation and reintegrated into society. Quality of life is assumed to increase logarithmically between stroke onset and 12 months post-stroke based on existing data from our phase II RCT, B-STARS. QALYs will be assessed by combining mortality and quality of life using an area-under-the-curve approach. The Dutch tariffs will be used to convert the EQ-5D-5L scores into a utility score. This utility score ranges from 0 to 1, in which 0 corresponds to death and 1 corresponds to perfect health.

7. Costs

7.1. Costs of the intervention

Costs of the intervention/procedure itself will be calculated with a bottom-up approach estimated with a mixed method approach. For instance, we will use financial information from the rehabilitation centres combined with estimates on the time spent by personnel on the interventional procedure. The estimate will include the following costs: purchase and maintenance of the equipment, direct and indirect costs of personnel and costs of facilities.

7.2. Clinical and outpatient rehabilitation costs

Other healthcare resource use will be assessed in two ways. Direct healthcare costs during clinical rehabilitation will be calculated by multiplying the length of stay of the patient and the estimated daily cost of clinical rehabilitation for that rehabilitation centre. Similarly, outpatient rehabilitation costs will be calculated by multiplying the duration of the patient's outpatient rehabilitation with the estimated daily costs of outpatient rehabilitation for that rehabilitation centre. The costs of outpatient rehabilitation are only available for patients who go to the same rehabilitation centre for clinical and outpatient rehabilitation. Missing data will be imputed.

7.3. Other healthcare costs and informal care

Healthcare costs and informal care costs after clinical rehabilitation will be collected through online administration of an adaptation of the iMTA Medical Consumption Questionnaire (iMCQ) at 6- and 12-months post stroke. Extrapolation will be used to calculate costs between 6- and 9-months post-stroke, as those data are not available due to a recall period of 3 months. The iMCQ will be used to measure all direct medical costs (consultations, hospitalizations), and direct non-medical costs (family care). Healthcare resource use will be multiplied with Dutch unit costs to determine healthcare costs. Unit

costs will be based on tariffs of the Dutch Healthcare Authority and/or as described in the Dutch Costing guideline.

7.4. Productivity costs

Productivity losses for individual patients will be collected using online administration of an adaptation of the iMTA Productivity Cost Questionnaire (iPCQ) at 6- and 12-months post-stroke. The iPCQ adaptation consists of two modules: lost productivity at paid work due to absenteeism and lost productivity at paid work due to presenteeism. Extrapolation will be used to calculate costs between discharge and 5 months and 6- and 11-months post-stroke, as those data are not available due to a recall period of 4 weeks.

8. Questionnaires

The Dutch versions of the iMTA Medical Consumption Questionnaire and the iMTA Productivity Cost Questionnaire are adapted to the B-STARS2 study population. The questionnaires are sent out digitally using an online platform to capture research data for clinical trials (CASTOR). The digital questionnaires are constructed to hide irrelevant questions based on previous answers, minimizing the survey completion time and increasing the response rate. The list of questions in the questionnaires is shown in Appendix A.

9. Change log

Version	Changes	Date
2	- Changed the time horizon of the primary analysis<br>- Added the EQ-5D at 6 months post-stroke<br>- Added an assumption on the change in quality of life between stroke onset and 12 months post-stroke	14-10-2024

10. References

Appendix A

Vragenlijst Zorggebruik

De vragen in deze vragenlijst gaan over uw zorggebruik sinds uw ontslag uit het revalidatiecentrum. Deze gegevens gebruiken we om te bepalen of TMS-behandeling leidt tot een vermindering van zorgkosten doordat patiënten mogelijk sneller herstellen, en daardoor minder zorg nodig hebben. Nadat u een vraag heeft beantwoord verschijnen er 3 puntjes naast de vraag. Door op de 3 puntjes te klikken verschijnt de optie om uw antwoord op een vraag te wissen.

De vragen in deze vragenlijst gaan over de zorg die u heeft ontvangen in de afgelopen 3 maanden.

- Bent u ontslagen uit het revalidatiecentrum? Dat wil zeggen: is uw klinische opnameperiode beëindigd?
- Op welke datum bent u ontslagen uit het revalidatiecentrum? Het gaat hierbij om de datum waarop de klinische opname is beëindigd. Het is mogelijk dat u na het ontslag nog voor poliklinische revalidatie in het revalidatiecentrum bent geweest.

De volgende vraag gaat over poliklinische revalidatie en zorg uit de eerste lijn. Poliklinische revalidatie vindt plaats in een revalidatiecentrum of een ziekenhuis, onder begeleiding van een revalidatieteam bestaande uit o.a. een revalidatiearts, fysiotherapeut, ergotherapeut en een maatschappelijk werker. Behandeling uit de 1ste lijn vindt plaats in een fysio- of ergotherapiepraktijk, onder begeleiding van een fysio- of ergotherapeut.

- Heeft u in de afgelopen 3 maanden poliklinische revalidatiebehandeling of therapie uit de 1ste lijn ontvangen in verband met uw beroerte?
- Welke situaties zijn op u van toepassing geweest in de afgelopen 3 maanden? U kunt meerdere opties selecteren.
    - Ik heb poliklinische revalidatiebehandeling gehad in een revalidatiecentrum of ziekenhuis
    - Ik heb therapie uit de 1ste lijn bij een fysio- of ergotherapiepraktijk gehad.

- Ontvangt u de poliklinische revalidatiebehandeling in hetzelfde centrum als waar u klinische revalidatiebehandeling heeft gehad?

De volgende vragen gaan alleen over behandelingen die u heeft ontvangen uit de 1ste lijn. Heeft u geen afspraken gehad, vul dan 0 in.

- Hoeveel afspraken had u in de afgelopen 3 maanden met een maatschappelijk werker in de 1ste lijn?
- Hoeveel afspraken had u in de afgelopen 3 maanden met een fysiotherapeut in de 1ste lijn?
- Hoeveel afspraken had u in de afgelopen 3 maanden met een ergotherapeut in de 1ste lijn?
- Hoeveel afspraken had u in de afgelopen 3 maanden met een psycholoog in de 1ste lijn?

De volgende vragen gaan over overig zorggebruik. Hierbij mag u alle afspraken die u met verschillende zorgverleners heeft gehad vermelden, ook afspraken die geen verband hadden met uw beroerte. Heeft u geen afspraken gehad, vul dan 0 in.

- Hoeveel afspraken had u in de afgelopen 3 maanden met een huisarts?
- Hoeveel afspraken had u in de afgelopen 3 maanden met een praktijkondersteuner (POH)?
- Hoeveel afspraken had u in de afgelopen 3 maanden met een bedrijfsarts (indien van toepassing)?
- Hoe vaak bent u in de afgelopen 3 weken op de spoedeisende eerste hulp van een ziekenhuis geweest?
- Hoe vaak bent u in de afgelopen 3 maanden met een ambulance naar het ziekenhuis gebracht?
- Had u in de afgelopen 3 maanden een afspraak met een arts bij de polikliniek van het ziekenhuis? U mag afspraken met de revalidatiearts meetellen zolang deze niet onderdeel waren van de poliklinische revalidatie.

- Bij welke artsen bent u in de afgelopen 3 maanden geweest? En hoe vaak? Bijvoorbeeld met een cardioloog.

De volgende vragen gaan over opname in het ziekenhuis. Hierbij gaat het ook om een eventuele ziekenhuisopname die geen verband had met uw beroerte.

- Heeft u in de afgelopen 3 maanden in het ziekenhuis gelegen? Het gaat hierbij om een ziekenhuisopname sinds uw ontslag uit het revalidatiecentrum.
- Hoe vaak heeft u in het ziekenhuis gelegen?
- Hoeveel dagen heeft u in het ziekenhuis gelegen? Heeft u meer dan 1 keer in het ziekenhuis gelegen, tel dan alle dagen bij elkaar op.

De volgende vragen gaan over thuiszorg. Dit kan thuiszorg zijn voor huishoudelijke hulp (stofzuigen), verzorging van uzelf (hulp bij douchen) of verpleging (verband omdoen)

- Heeft u in de afgelopen 3 maanden hulp van de thuiszorg gehad? Dit kan thuiszorg zijn voor huishoudelijke hulp, verzorging van uzelf of verpleging.
- Hoeveel weken heeft u deze thuiszorg gehad? Tel alle weken bij elkaar op. Hierboven ziet u het maximale aantal weken dat u in kunt vullen.
- Hoeveel uur thuiszorg kreeg u in deze weken gemiddeld per week?

De volgende vragen gaan over dagbehandeling bij een woon-/zorgcentrum.

- Bent u in de afgelopen 3 maanden in dagbehandeling geweest bij een woon-/zorgcentrum?
- Hoe vaak moest u naar het woon-/zorgcentrum?

De volgende vragen gaan over verblijf in een zorginstelling.

- Bent u in de afgelopen 3 maanden in een woon-/zorgcentrum blijven slapen?
- Hoelang bent u in het woon-/zorgcentrum geweest? Bent u er meer dan 1 keer geweest, tel dan alle dagen bij elkaar op.

De volgende vragen gaan over hulp van familieleden en naasten die u misschien ontvangt vanwege de gevolgen van uw beroerte. Dit kan huishoudelijke hulp (stofzuigen,

boodschappen doen of verzorgen van kinderen), verzorging van uzelf (hulp bij douchen of hulp bij eten en drinken) of praktische hulp (ondersteuning bij wandelen of u ergens heen brengen) zijn.

- Heeft u in de afgelopen 3 maanden hulp gekregen van een familielid of naaste vanwege de gevolgen van uw beroerte? Bijvoorbeeld huishoudelijke hulp, verzorging van uzelf of praktische hulp?
- Hoeveel weken heeft u deze hulp gehad? Tel alle weken bij elkaar op. Hierboven ziet u het maximale aantal weken dat u in kunt vullen.
- Hoeveel uur hulp kreeg u in deze weken gemiddeld per week?

Vragenlijst productiviteit

De vragen in deze vragenlijst gaan over betaald werk dat u deed voor de beroerte, en over eventuele terugkeer naar werk. Deze gegevens gebruiken we om te bepalen of TMS-behandeling leidt tot een vervroegde terugkeer naar werk.

De volgende vragen gaan over betaald werk.

- Verrichtte u voor uw beroerte betaald werk?
- Hoeveel uur per week werkte u?
- Op hoeveel dagen in de week werkte u?
- Heeft u de afgelopen 4 weken betaald werk verricht? Het gaat hierbij ook om werk in het kader van re-integratie.
- Wanneer bent u (weer) begonnen met betaald werk?
- Doet u hetzelfde werk als voor uw beroerte?
- Hoeveel uur per week heeft u betaald werk verricht in de afgelopen 4 weken? Het gaat hierbij om het gemiddeld aantal uur per week dat u betaald werk heeft verricht. Het aantal uren dat u afwezig bent geweest door re-integratie of ziekte tellen niet mee.
- Waren er in de afgelopen 4 weken dagen waarop u wel gewerkt heeft maar tijdens uw werk last had van de gevolgen van uw beroerte?

- Op hoeveel werkdagen had u tijdens uw werk last van de gevolgen van uw beroerte in de afgelopen 4 weken? Tel alleen de werkdagen bij elkaar op.
- Op de dagen dat u last had, kon u misschien niet zoveel werk doen als normaal. Hoeveel werk kon u op deze dagen gemiddeld doen op een schaal van 0 tot 10?
